# Genetic parameters and genomic prediction accuracy for piling behaviour using individual-level computer-vision data from ArUco-marked laying hens

**DOI:** 10.1186/s12711-026-01109-x

**Published:** 2026-11-12

**Authors:** T. Osorio-Gallardo, A. van Putten, D. Janssen, C. K. Annene, T. B. Rodenburg, P. Bijma

**Affiliations:** 1 Wageningen University and Research, Droevendaalsesteeg 4, Wageningen, 6708 PB Netherlands; 2 Utrecht University, Heidelberglaan 8, Utrecht, 3584 CS Netherlands; 3 Hendrix Genetics, Spoorstraat 69, Boxmeer, 5831 CK Netherlands; 4 Aarhus University, Nordre Ringgade 1, Aarhus Centrum, 8000 Denmark

## Abstract

**Background:**

Recurring piling, a detrimental behaviour in laying hens where birds cluster densely, leads to heat stress, injuries, and potentially smothering. While its occurrence is a major concern in large group housing systems for laying hens, the causes are poorly understood, and management interventions have shown variable success. Line differences suggest a genetic predisposition, but the absence of individual-level phenotypic data has, until now, prevented a formal genetic analysis. This study aimed to estimate, for the first time, the genetic parameters and genomic breeding values of piling behaviour in laying hens.

**Results:**

We monitored 1,441 genotyped Dekalb White hens at 40 weeks of age in a semi‑industrial facility over nine days. Individual participation in 115 piling events was recorded using automated video tracking with ArUco markers. Three traits were defined: overall participation in piles (OSI; binary), event-specific participation (OSE; binary), and a continuous rank score based on joining order (RSE). Genomic relationship matrices were used to estimate variance components, heritability, and prediction accuracy. Significant genetic variance was found for all traits. Heritability estimates on the observed scale were low to moderate: 0.11 for OSI, 0.05 for OSE, and 0.05 for RSE. Cross‑validation accuracies of genomic breeding values ranged from 0.27 to 0.44. Selection against the lowest 10% of estimated breeding values was predicted to reduce trait expression by 21–42%, indicating good opportunities for genetic improvement. Genetic correlations among traits were high (point estimates > 0.99). The most accurate genomic predictions were obtained by using event‑level data (OSE) to predict the overall propensity to pile (OSI).

**Conclusions:**

This study provides the first evidence that piling behaviour in laying hens is a heritable trait. The significant genetic variance and predicted response to selection across traits confirm the potential for piling mitigation through genetic selection. A practical strategy for breeding programs could involve selecting against the overall propensity to pile (OSI) using event-level participation data (OSE). These findings open a new avenue for mitigating piling through breeding.

**Supplementary Information:**

The online version contains supplementary material available at https://doi.org/10.1186/s12711-026-01109-x.

## Background

In response to growing societal concern for the welfare of laying hens in egg production, the use of conventional cages was banned in the EU as of January 2012, and the use of alternative housing systems (furnished and non-cage systems) was made mandatory [1]. In North-West Europe, this mainly led to a transition to non-cage systems, where hens are housed in large groups. These housing systems allowed hens to express natural behaviours such as nesting, perching, and foraging. However, these systems could also be associated with the expression of detrimental behaviours such as piling and smothering [2].

Piling occurs when hens crowd together in densely packed groups, and smothering refers to any mortality resulting from a piling event. Piling may result in smothering, but mortality is not always a consequence of piling [3, 4]. Recurring piling is a distinct type of piling behaviour. Unlike acute panic piling, which is triggered by sudden stressors, recurring piling presents itself gradually and often without a clear stimulus, reappearing in the same zones across peaking periods [4]. Available evidence indicates that recurrent piling and smothering events are most frequently reported during the laying period, particularly around peak production or shortly thereafter, rather than during early rearing or late lay [4, 5]. While these piling events tend to involve calm clustering rather than frantic movement, they can contribute cumulatively to heat stress, injuries, diminished egg production, and occasional mortality.

Despite awareness of recurring piling, its causes remain unclear, and management interventions are often reactive and labour-intensive. Common strategies (e.g. altering lighting regimes, temporary flock disruption, or housing enrichments) are ad hoc and often ineffective at preventing recurrence [6]. Hypothesized drivers for piling include attraction to light, environmental gradients, comfort-seeking, fear, or stress [5, 7]. However, increasing evidence points also to a possible genetic basis for piling motivation: for example, higher frequencies and longer durations of piling have been reported in brown-egg layer flocks compared to white-egg flocks, suggesting breed-related predispositions [6]. Furthermore, behavioural traits such as fearfulness and social responsiveness, which can influence piling behaviour, have been shown to possess a heritable component in laying hens [8]. However, no study to date has formally estimated genetic parameters for piling behaviour.

One likely reason for the absence of genetic studies on piling is the lack of individual-level phenotypic data. Piling often occurs in large groups (groups consisting of thousands of hens), making it difficult to identify and track individual involvement through observation alone. As a result, most reports on piling have been based on group-level metrics, which are insufficient for quantitative genetic analysis. However, recent advances in computer vision technology now offer the possibility to generate individual-level tracking data within groups of commercial laying hens (e.g [9, 10]). By combining individual-level behavioural data from automated tracking with genomic information, we can now estimate genetic parameters for behaviours on which genetic analysis was previously impossible.

Here we present, to our knowledge, the first estimates of variance components and heritabilities, and of genomic breeding values and their accuracy for piling behaviour in laying hens. This offers novel insight into the genetic basis of piling behaviour, and quantifies the potential for genetic selection to reduce its occurrence.

## Methods

### Raw data

A total of 1,441 Dekalb White laying hens were provided by Hendrix Genetics^®^ (HG) for this study. All birds were of the same age and originated from the same hatchery. The population consisted of three-way crossbred hens, produced by inseminating crossbred dams (maternal line) with pooled semen from pureline sires. Each bird was genotyped using the HG and Cobb-Vantress custom-made Illumina 60 K SNP chip for layers, and the genotypes were also provided by HG.

**Fig. 1:**
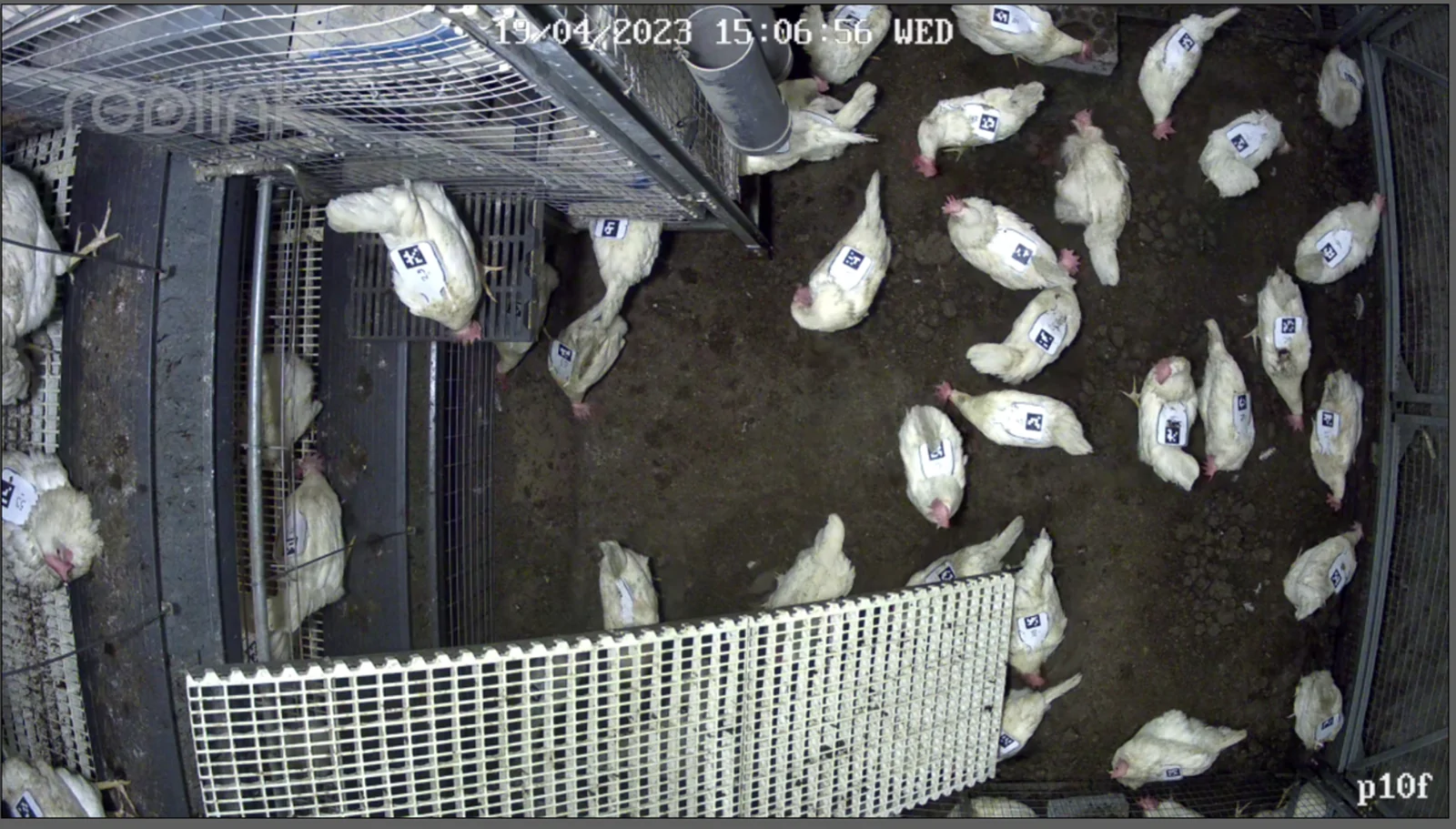
Example of the field of view of the camera. The entrance to the pen is on the right, and the upper levels are on the left. Adjacent pens are at the top and bottom of the image, with the ramp located at the bottom

At approximately 20 weeks of age, birds were transferred to a commercial-style facility and distributed across 12 pens located within a single barn. Each pen measured 195 cm × 203 cm and housed approximately 120 hens. The pens were structured into three vertical levels: a litter area on the ground, a nesting area, and an elevated perching area, with a total usable area of approximately 9 m squared. Access to upper levels was facilitated by a ramp from the litter area to the top tier, reducing the risk of injury during movement between levels (Fig. 1). Birds were maintained under a commercial 16 L:8D lighting schedule with a light intensity of approximately 25 lx. Ambient temperature was maintained at approximately 20 °C. A small amount of wood shavings was provided when birds were first housed, after which litter accumulated through the addition of dry manure to a depth of approximately 5 cm. Feed was provided through multiple daily runs of a chain feeder during the light period. Information on relative humidity was not available.

**Fig. 2:**
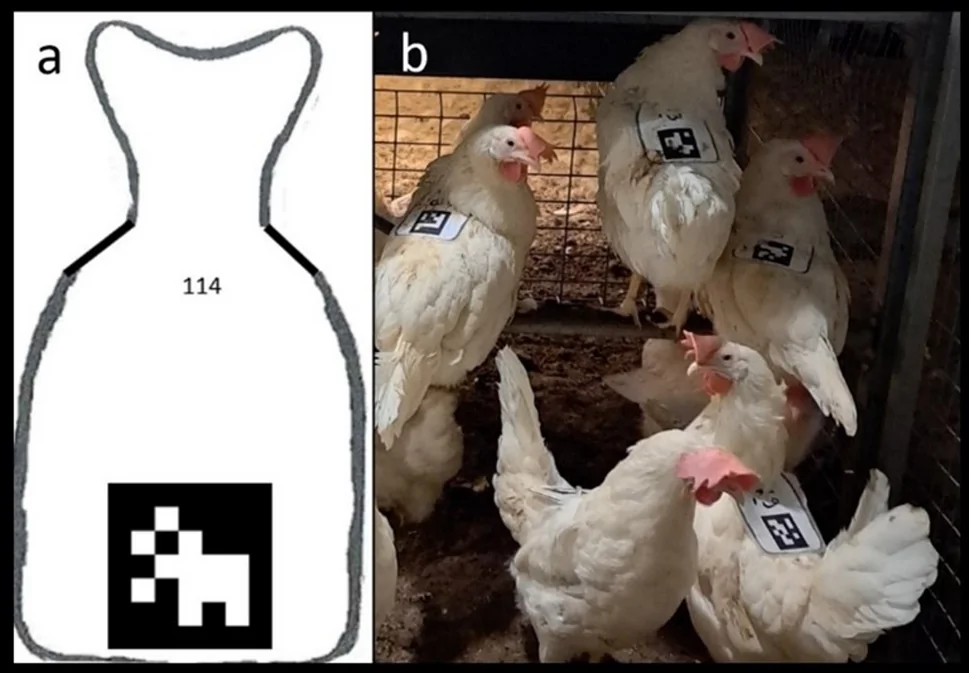
Example of backpack and backpack placement. (**a**) sheet of paper with backpack design and ArUco marker number 114; (**b**) example of backpacks after placement

To enable individual identification for video-based tracking, each hen was fitted with a unique ArUco marker. A set of 150 markers was generated using the 6 × 6 dictionary OpenCV library [11]. Each marker was printed onto a pre-designed backpack template (Fig. 2a), which was laminated, cut, and fitted with elastic straps for ergonomic placement. Sixteen weeks after pen housing (approximately 36 weeks of age), and once a nutritional trial that used the same hens had ended, the backpacks were placed on all individuals (Fig. 2b). Each bird’s wing-tag number was matched with its ArUco marker ID, and this information was logged. Markers were unique within each pen but could be repeated across pens. Camera positioning minimized the risk of detecting markers from adjacent pens.

For recording, one surveillance camera (B800, Reolink, USA) was installed above the litter area of each pen (Fig. 1), with no visual access to the nesting or perching levels. Thus, hens were recorded exclusively while in the litter zone. Continuous video footage was collected during day time over nine days, capturing approximately 11 h per day per pen. Video resolution was set to 2304 × 1296 pixels at 20 frames per second.

### Piling event annotation

**Fig. 3:**
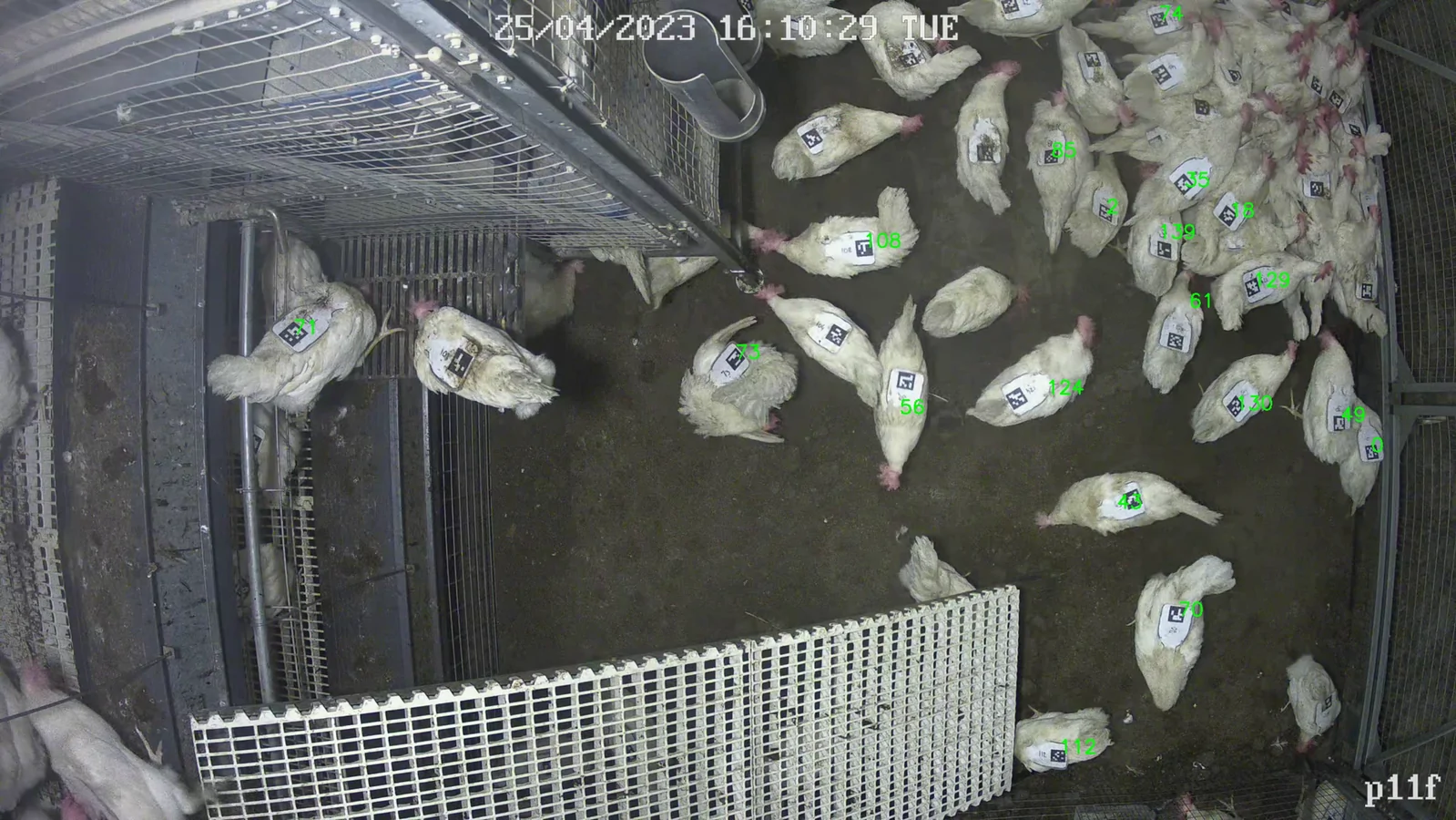
Example of a piling event detected after the video was processed by the script

For this study, a piling event was defined as a clustering of at least five individuals physically converging in close proximity, with sustained contact and piling-like behaviour (e.g. climbing over each other, compressing against pen boundaries, or forming a dense, immobile group, Fig. 3). Piling events were first detected using a custom computer vision script written in Python [12], which identified relevant events across the full video dataset. When a piling event was detected, the script automatically cropped the corresponding video segment, which started one minute before the event and continued until the pile had fully dissolved. For each frame within this cropped segment, the script overlaid the detected ArUco marker IDs directly above the individuals they belonged to (Fig. 3).

Up to 20 participating birds per event were manually annotated based on their participation sequence. Beyond this number, reliable tracking of individual participants became increasingly difficult because piling events were highly dynamic, with hens frequently entering, leaving, and repositioning within the pile. Larger piles may have occurred, but total pile size beyond the first 20 individuals was not quantified. The smallest annotated pile consisted of five individuals. For each piling event, these birds were recorded in the order they joined the pile. The ArUco ID, time of entry (recorded to the nearest millisecond), and joining rank were saved to a .csv file.

### Final data

All editing and statistical analysis were performed using scripts written in the R 4.4.1 programming language [13].

Table 1 presents a summary of the final dataset used for genetic analysis of piling behaviour. The total number of individual participations exceeded the number of unique participants, indicating that several individuals participated in multiple piling events. This repeated participation suggests consistent individual behavioural tendencies and potential genetic influences on piling behaviour across events. In addition, the total number of participating unique individuals is less than half of the total number of individuals, which means that more than half of the animals did not participate in the piling events.

**Table 1:** Summary of the final dataset used for genetic analysis

Total number of days	9
Total number of pens	12
Total number of piling events	115
Total number of individuals	1432
Total number of piling participations	1606
Total number of participating unique individuals	694

### Traits

The phenotypes analysed in this study were designed to measure an individual’s propensity to participate in piling events. This approach treats piling events as opportunities for participation and focuses on the behavioural response of individual hens once a piling event has started.

Three traits were derived to describe individual involvement in piling events: two binary traits and one continuous trait. Each genotyped individual received a score for each trait. The traits were defined as follows:

- One Score per Individual (OSI) – Binary Trait: Each individual received a score of “1” if it participated in at least one piling event across the entire observation period. Individuals that were never annotated as participants were scored as “0”.
- One Score per Event (OSE) – Binary Trait: Each individual housed in the pen where the piling event occurred received a score for that specific event. If the individual was annotated as a pile participant, the score was “1”; if not, “0”. This resulted in repeated measurements per individual, depending on the number of piling events that occurred in its pen.
- Rank Score per Event (RSE) – Continuous Trait: This trait considers how early an individual joined a pile on a 0 to 1 scale. A bounded 0–1 scale was used to represent relative joining order within each piling event, facilitating comparison across events of different sizes and with other traits. Initiators of the piles were assigned a score of “1”. For the remaining annotated participants, the score was calculated as:

1 $$1 - \left(\frac{\mathrm{rank}- 1}{\max\text{rank of pile}}\right)$$

where *rank* represents the order number in which the individual joined the pile, and *max rank of pile* is the joining rank of the last participant. For example, for a pile with 10 participants, RSE takes values from 1 to 0.1. Thus, the first joining individual had an RSE = 1, the last had an RSE = 0.1, and, for example, the eighth joining individual had an RSE = 0.3. All other individuals were assigned a score of “0”. Like OSE, this trait included repeated observations per individual.

### Genetic analysis

All individuals were genotyped using a custom Illumina 60 K SNP chip co‑developed by HG and Cobb‑Vantress for layers. As no pedigree information was available, a genomic relationship matrix (GRM) was calculated. Before GRM construction, genotype quality control (QC) was performed using PLINK 1.9 [14]. We retained 44,813 SNPs located on autosomal chromosomes, after applying filters for a minor allele frequency (MAF) > 0.01 and a genotyping rate > 0.90 per SNP. No Hardy–Weinberg equilibrium filter was applied, as deviations from equilibrium are expected in crossbred populations. The GRM was calculated from the QC-pruned genotypes using VanRaden’s first method [15] as implemented in the calc_grm software [16]. The estimation of variance components, genetic parameters, and breeding values was performed using ASReml 4.2.1 software [17].

Because most piling events were unique to a given pen–day–hour combination, the effects of pen, day, hour, and event number represented largely overlapping sources of variation. In other words, event number already captured most of the information contained in the other terms. Therefore, for the event-level traits OSE and RSE, the event number (e.g., pile #1, pile #2, etc.) was retained as the sole fixed effect and fitted as a categorical factor to avoid redundancy. For the individual-level trait (OSI), pen was included as a fixed effect to account for differences among pens across the observation period, while event number was omitted because this trait condensed piling tendency across all recorded events rather than specific ones.

### Estimation of variance components

#### Threshold models for binary traits

Univariate animal threshold models were used to estimate the genetic parameters of both binary traits, OSI and OSE. These threshold models were implemented as Generalized Linear Mixed Models (GLMM), using a probit link function and a binomial distribution with *n* = 1. In both cases, the binary observed phenotype * y* was assumed to be a discretized expression of an underlying continuous liability $\:{y}^{*}$, that followed a linear mixed model.

#### Model for OSI

The model included a fixed pen effect and a random genetic effect. The liability model for individual i was:

2 $$\:{\mathrm{y}}_{{1}_{\mathrm{i}}}^{\mathrm{*}}=\:{\upmu\:}+{\mathrm{P}\mathrm{e}\mathrm{n}}_{\mathrm{i}}+{\mathrm{a}}_{\mathrm{i}},$$

where $\:{\upmu\:}$ is the overall mean of the trait, $\:{\mathrm{P}\mathrm{e}\mathrm{n}}_{\mathrm{i}}$ is the fixed effect of the housing pen (1–12), $\:{\mathrm{a}}_{\mathrm{i}}$ is the genetic effect of the individual with distribution $\:\mathbf{a}\:\sim\:\mathrm{N}\left(0,{\mathbf{G}{\upsigma\:}}_{\mathrm{a}}^{2}\:\right)$, where $\:\mathbf{a}$ is a vector of genetic effects, $\:{{\upsigma\:}}_{\mathrm{a}}^{2}$ is the genetic variance, and **G** is the GRM. While a GLMM has no residual in the liability scale, the implicit residual variance corresponding to the probit link function is equal to one.

#### Model for OSE

For OSE, in addition to the genetic effect, a permanent environmental effect for the individual was included as a random effect to account for repeated observations across events. The event number was fitted as a fixed effect to correct for environmental variation between events (see Discussion). The liability model for individual i in event j was:

3 $$\:{\mathrm{y}}_{{\mathrm{2}}_{\mathrm{ij}}}^{\mathrm{*}}\text=\mu+{\mathrm{Event}}_{\mathrm{j}}\mathrm{+}{\mathrm{a}}_{\mathrm{i}}\mathrm{+}{\mathrm{p}}_{\mathrm{i}},\:$$

where $\:{\mathrm{E}\mathrm{v}\mathrm{e}\mathrm{n}\mathrm{t}}_{\mathrm{j}}$ is the fixed effect of event number, $\:{\mathrm{p}}_{\mathrm{i}}$ is the permanent environmental effect of the animal with variance $\:{{\upsigma\:}}_{p}^{2}$ and assumed distribution $\:\mathbf{p}\:\sim\:\mathrm{N}\left(0,\mathbf{I}{{\upsigma\:}}_{p}^{2}\:\right).$ $\:\mathbf{I}$ denotes an identity matrix, and other terms are as previously defined.

#### Linear mixed model for RSE

As for OSE, event number was fitted as a fixed effect to account for environmental variation between events. Let $\:{\mathrm{y}}_{{3}_{\mathrm{i}\mathrm{j}}}$ represent the observed continuous phenotype for RSE of individual i in event number j. The following linear mixed animal model was used:

4 $$\:{\mathrm{y}}_{{3}_{\mathrm{i}\mathrm{j}}}^{}=\:{\upmu\:}+{\mathrm{E}\mathrm{v}\mathrm{e}\mathrm{n}\mathrm{t}}_{\mathrm{j}}+{\mathrm{a}}_{\mathrm{i}}+{\mathrm{p}}_{\mathrm{i}}+\:{\mathrm{e}}_{\mathrm{i}\mathrm{j}},\:$$

where $\:{\mathrm{E}\mathrm{v}\mathrm{e}\mathrm{n}\mathrm{t}}_{\mathrm{j}}$ is the fixed effect of event number, $\:{\mathrm{e}}_{\mathrm{i}\mathrm{j}}$ is a normally distributed residual, and the other effects and their distributions are as previously described.

#### Heritability

For each trait, heritability and its standard error were estimated with ASReml using the formula:

5 $$\:{\mathrm{h}}^{2}=\:\frac{\widehat{{{\upsigma\:}}_{\mathrm{a}}^{2}}}{\widehat{{{\upsigma\:}}_{\mathrm{a}}^{2}}+\:\widehat{{{\upsigma\:}}_{\mathrm{p}}^{2}}+\widehat{{{\upsigma\:}}_{\mathrm{e}}^{2}}},$$

where $\:\widehat{{{\upsigma\:}}_{\mathrm{a}}^{2}}$ is the estimated genetic variance, $\:\widehat{{{\upsigma\:}}_{\mathrm{p}}^{2}}$ the estimated variance of the permanent environmental effect and $\:\widehat{{{\upsigma\:}}_{\mathrm{e}}^{2}}$ the estimated residual variance. For the OSI trait, which did not include a permanent environmental effect, $\:\widehat{{{\upsigma\:}}_{\mathrm{p}}^{2}}$ was omitted.

For the binary traits OSI and OSE, heritability was initially estimated on the liability scale because variance components were estimated using a GLMM. In these models, the residual variance was fixed at 1.

To facilitate interpretation, the heritability estimates for OSI and OSE were then transformed to the observed scale, following [18]:

6 $$\:{\mathrm{h}}_{\mathrm{o}\mathrm{b}\mathrm{s}}^{2}=\:\frac{{\mathrm{h}}_{\mathrm{l}\mathrm{i}\mathrm{a}\mathrm{b}}^{2}\:{\mathrm{z}}^{2}}{{\uppi\:}\left(1-{\uppi\:}\right)},$$

where $\:{\mathrm{h}}_{\mathrm{o}\mathrm{b}\mathrm{s}}^{2}$ is the heritability on the observed scale, $\:{\mathrm{h}}_{\mathrm{l}\mathrm{i}\mathrm{a}\mathrm{b}}^{2}$ is the heritability on the liability scale, $\:{\uppi\:}$ is the proportion of individuals with phenotype = 1, and $\:\mathrm{z}$ is the height of the standard normal density at the threshold corresponding to $\:{\uppi\:}$. Standard errors of $\:{\mathrm{h}}_{\mathrm{o}\mathrm{b}\mathrm{s}}^{2}$ were obtained by applying the same transformation to the corresponding liability-scale standard errors reported by ASReml.

The model for RSE directly yields estimates on the observed scale, so no transformation was applied there.

#### Simulation study

A simulation study was performed to evaluate whether the OSI and OSE models recovered known values of genetic variance and heritability under a simple data-generating process. Simulated data were generated in R and analysed using ASReml.

The true genetic variance and residual variance were set to 0.25 and 1, respectively, resulting in an expected heritability of 0.20. The **G** used in the simulation was the same as that used for the estimation of genetic parameters in the study population. Genetic effects were simulated as $\:\mathbf{a}\:\sim\:\mathrm{N}\left(0,\:0.25\mathbf{G}\right)$, and residual effects were simulated as $\:\mathbf{e}\:\sim\:\mathrm{N}\left(0,\mathbf{I}\:\right)$. A continuous phenotype on the liability scale was generated according to:

7 $$\:\mathbf{y}\:=\:\mathbf{Z}\mathbf{a}+\mathbf{e},$$

where **Z** is the incidence matrix linking observations to individuals. Ten repeated observations were simulated for each hen.

To mimic the structure of the OSE phenotype, individuals were randomly assigned to 12 pens and a unique event identifier was generated for each pen-by-event combination, resulting in 120 simulated events (12 pens × 10 events). The simulated continuous phenotype was converted to a binary phenotype using a threshold of 1, where each simulated observation with a value below 1 was assigned 0 and each observation with a value greater than or equal to 1 was assigned 1. The OSI phenotype was subsequently constructed by collapsing the repeated simulated OSE observations into a single binary outcome indicating whether an individual had at least one simulated OSE observation with a value of 1.

One hundred simulation replicates were generated. Each replicate was analysed using four different models:

- Animal model for the simulated continuous phenotype:
  This was the same linear mixed model used to generate the data, Eq. (7) and was fitted to verify recovery of the simulated genetic variance and heritability.

- Simplified threshold model for simulated OSE phenotypes:
  This GLMM, fitted using a probit link function, was the binary threshold-model equivalent of the data-generating model in Eq. (7). It was used as a benchmark to evaluate how converting the simulated continuous phenotype to a binary phenotype affected recovery of the simulated genetic variance and heritability.

- Complete threshold model for simulated OSE phenotypes:
  This was the same model used for variance component estimation of OSE in the present study, Eq. (3).

- Threshold model for simulated OSI phenotypes:
  This was the same model used for variance component estimation of OSI in the present study, Eq. (2).

Heritability for the continuous model was calculated as: $\:{\mathrm{h}}^{2}=\:\frac{\widehat{{{\upsigma\:}}_{\mathrm{a}}^{2}}}{\widehat{{{\upsigma\:}}_{\mathrm{a}}^{2}}+\widehat{{{\upsigma\:}}_{\mathrm{e}}^{2}}}$

For the threshold models, heritability was calculated as: $\:{\mathrm{h}}^{2}=\:\frac{\widehat{{{\upsigma\:}}_{\mathrm{a}}^{2}}}{\widehat{{{\upsigma\:}}_{\mathrm{a}}^{2}}+\:\widehat{{{\upsigma\:}}_{\mathrm{p}}^{2}}+\widehat{{{\upsigma\:}}_{\mathrm{e}}^{2}}}$, where $\:\widehat{{{\upsigma\:}}_{\mathrm{p}}^{2}}$ was omitted for models that did not include a permanent environmental effect. For all threshold models, the residual variance was fixed to 1 because a probit link function was used.

### Potential for genetic improvement

To assess the practical significance of the estimated genetic parameters and genomic breeding values, the expected response to selection was estimated assuming selection of the 10% of individuals with the lowest EBVs. According to the breeder’s equation [19], the expected response to selection can be expressed as R=iρσ_A_, where i is the selection intensity, ρ is the accuracy of the selection criterion, and σ_A_ is the additive genetic standard deviation. When selection is based on EBVs, the product ρσ_A_ corresponds to the standard deviation of the EBVs, and the response to selection can therefore be expressed as R=iσ_EBV_. The term iσ_EBV_ is equivalent to the selection differential on the EBV scale. Therefore, the expected response to selection can be calculated directly from the difference between the mean EBV of the selected individuals and the mean EBV of the entire population. For the binary traits (OSI and OSE), response was defined as the expected change in prevalence. For the continuous trait (RSE), response was defined as the expected change in the population mean RSE following selection.

- Binary traits:

For OSI and OSE, response on the liability scale ($\:{\mathrm{R}}_{\mathrm{l}\mathrm{i}\mathrm{a}\mathrm{b}})$ was obtained

8 $$R_{\mathrm{liab}} = \overline{\mathrm{EBV}}_{10\%} - \overline{\mathrm{EBV}}_{\mathrm{all}}$$

where $\overline{\mathrm{EBV}}_{\mathrm{all}}$ is the mean liability EBV for the full population and $\overline{\mathrm{EBV}}_{10\%}$ is the mean liability EBV of the lowest 10% of individuals. To translate the response to selection from the liability scale to the observed scale (proportion expressing the trait), the initial population prevalence ($\:{\uppi\:}$) was converted to the corresponding threshold on the liability scale: $\:\mathrm{t}={{\Phi\:}}^{-1}(1-{\uppi\:}$) where $\:\:{{\Phi\:}}^{-1}$ is the inverse cumulative distribution function of the standard normal distribution. The new threshold after selection was calculated as: $\:{\mathrm{t}}^{{\prime\:}}=\mathrm{t}-\:{\mathrm{R}}_{\mathrm{l}\mathrm{i}\mathrm{a}\mathrm{b}}\:$ and the new expected prevalence was obtained by back-transforming to the observed scale: $\:{{\uppi\:}}^{{\prime\:}}=1-\:{\Phi\:}\left({\mathrm{t}}^{{\prime\:}}\right)$.

- Continuous trait:

For RSE, the expected response was calculated directly from the selection differential in $\overline{\mathrm{EBV}}$

9 $$\:{\mathrm{R}}_{\mathrm{R}\mathrm{S}\mathrm{E}}= \overline{\mathrm{EBV}}_{10\%} - \overline{\mathrm{EBV}}_{\mathrm{all}},$$

### Cross-validation and genomic prediction accuracy

To assess the quality of genomic EBVs, we calculated both model-based accuracies and accuracy and dispersion of EBV based on cross-validation. The cross-validation accuracy of the EBVs for all traits was estimated using a leave-one-pen-out cross-validation approach. In this design, the phenotypic information of the individuals housed in each pen was sequentially omitted from the dataset while information of the individuals in other pens remained. The phenotypic information of the individuals in other pens was then used to calculate EBVs for the individuals with masked phenotypes (EBVMPs). For each fold, the model specified for the respective trait (see Genetic Analysis section) was fitted to estimate the EBVMPs. This process was repeated, each time masking a different pen, until each pen had been masked once.

#### Model-based EBVMP accuracy

The average reported accuracy for each trait was calculated using the standard errors of the individual EBVMPs obtained, as reported by ASReml in the .sln output file [20].

10 $$\:{{\uprho\:}}_{\widehat{\mathrm{a}}}=\mathrm{a}\mathrm{v}\mathrm{e}\mathrm{r}\mathrm{a}\mathrm{g}\mathrm{e}\left(\sqrt{\frac{\widehat{{{\upsigma\:}}_{\mathrm{a}}^{2}}-\:{\mathrm{S}\mathrm{E}}_{\mathrm{i}}^{2}}{\widehat{{{\upsigma\:}}_{\mathrm{a}}^{2}}}}\right),$$

where $\:{{\uprho\:}}_{\widehat{\mathrm{a}}}$ is the accuracy of EBVMP averaged over individuals, $\:\widehat{{{\upsigma\:}}_{\mathrm{a}}^{2}}$ is the estimated genetic variance of the trait, and $\:{\mathrm{S}\mathrm{E}}_{\mathrm{i}}$ the standard error of the EBVMP of individual i as reported by ASReml. Note that this reported accuracy refers to individuals without own performance records.

#### Validation model and estimation of regression coefficient

Next, a validation model was fitted to the full dataset. This model was identical to the original genetic model for a given trait, with two key modifications: the genetic effect was removed, and the respective EBVMPs were included as a fixed covariate. For example, the validation model for RSE was:

11 $$\:{y}_{{3}_{ij}}=\:{\upmu\:}+{\mathrm{E}\mathrm{v}\mathrm{e}\mathrm{n}\mathrm{t}}_{\mathrm{j}}+{\mathrm{E}\mathrm{B}\mathrm{V}\mathrm{M}\mathrm{P}}_{\mathrm{i}}\mathrm{*}{\upbeta\:}+{\mathrm{p}}_{\mathrm{i}}+\:{\mathrm{e}}_{\mathrm{i}\mathrm{j}},$$

where EBVMP is a covariate for the animal’s EBV from masked phenotypes with regression coefficient $\:{\upbeta\:}$ estimated as a fixed effect. For proper BLUP EBVs, the regression coefficient of masked phenotypes on EBVs is equal to one. Therefore, the dispersion of EBVMP was measured by the estimate of β, where $\:\widehat{{\upbeta\:}}$ =1 indicates correct dispersion, $\:\widehat{{\upbeta\:}}$ < 1 overdispersion of EBV, and $\:\widehat{{\upbeta\:}}\:$> 1 underdispersion of EBV (e.g [21, 22]), .

The cross-validation accuracy of EBVMPs $\:\left({{\uprho\:}}_{\mathrm{E}\mathrm{B}\mathrm{V}\mathrm{M}\mathrm{P}}\right)$, i.e., the correlation between EBVMP and the true breeding value, was estimated using the relationship:

12 $$\:{{\uprho\:}}_{\mathrm{E}\mathrm{B}\mathrm{V}\mathrm{M}\mathrm{P}}=\frac{{\uprho\:}\:\left(\mathrm{E}\mathrm{B}\mathrm{V}\mathrm{M}\mathrm{P},\:\stackrel{\sim}{\mathrm{y}}\right)}{\mathrm{h}},$$

where ρ(EBVMP, $\:\stackrel{\sim}{\mathrm{y}}$) is the correlation between the EBVMPs and the phenotypic observations corrected for fixed effects ($\:\stackrel{\sim}{\mathrm{y}}$), and h is the square root of the trait’s heritability. Similar approaches for estimating prediction accuracy have been used in previous studies (e.g [23, 24]).

The $\:{\uprho\:}\:(\mathrm{E}\mathrm{B}\mathrm{V}\mathrm{M}\mathrm{P},\stackrel{\sim}{\mathrm{y}})$ was calculated as the square root of the proportion of variance in $\:\stackrel{\sim}{\mathrm{y}}$ explained by the regression on EBVMPs. Thus, the accuracy of EBVMPs was estimated as

13 $$\:{{\uprho\:}}_{\mathrm{E}\mathrm{B}\mathrm{V}\mathrm{M}\mathrm{P}}=\frac{\sqrt{\frac{{\widehat{{\upbeta\:}}}^{2}{{\upsigma\:}}_{\mathrm{E}\mathrm{B}\mathrm{V}\mathrm{M}\mathrm{P}}^{2}}{{\widehat{{\upbeta\:}}}^{2}{{\upsigma\:}}_{\mathrm{E}\mathrm{B}\mathrm{V}\mathrm{M}\mathrm{P}}^{2}+\:\widehat{{{\upsigma\:}}_{p}^{2}}+\widehat{{{\upsigma\:}}_{\mathrm{e}}^{2}}}}}{\mathrm{h}},$$

where the numerator represents the square root of the proportion of variance in $\:\stackrel{\sim}{\mathrm{y}}$ explained by EBVMP, $\:\widehat{{\upbeta\:}}$ the regression coefficient estimated with the validation model, $\:{{\upsigma\:}}_{\mathrm{E}\mathrm{B}\mathrm{V}\mathrm{M}\mathrm{P}}^{2}$ the variance of the EBVs of masked phenotypes, and hats denote estimates. The heritability values used in the denominator were those estimated on the same scale as the respective EBVMPs (i.e., liability scale for OSI and OSE, and observed scale for RSE). In the case of OSI, $\:\widehat{{{\upsigma\:}}_{\mathrm{p}}^{2}}$ was removed from the formula as this component was not estimated. For OSI and OSE, $\:\widehat{{{\upsigma\:}}_{\mathrm{e}}^{2}}$ = 1. For all traits, the values of $\:\widehat{{{\upsigma\:}}_{\mathrm{p}}^{2}}$ and $\:\widehat{{{\upsigma\:}}_{\mathrm{e}}^{2}}$ used in the formula were those obtained with the validation models. For more information on the derivation of this formula look at Appendix A.

#### Between-trait genomic prediction accuracy and genetic correlation

The principle that genetically correlated traits can enhance genomic prediction is well-established [25, 26]. Specifically, if the same genomic regions influence multiple traits, then breeding values for one trait can contain predictive information for another.

Here, we used between-trait cross-validation for two purposes: first, to investigate whether EBVs for one trait also predict the other trait, and second, to estimate the genetic correlations between the traits. This strategy was used because attempts to perform pair-wise bivariate trait analysis in ASREML failed to converge.

Therefore, we extended our validation procedure to all pairwise trait combinations. In this analysis, EBVMPs estimated for one trait were used as a predictor in the validation model for another trait. For example, when predicting OSE with EBVMPs for OSI, the model was:

14 $$\:{y}_{{2}_{ij}}=\:{\upmu\:}+{\mathrm{E}\mathrm{v}\mathrm{e}\mathrm{n}\mathrm{t}}_{\mathrm{j}}+{\mathrm{O}\mathrm{S}{\mathrm{I}}_{\mathrm{E}\mathrm{B}\mathrm{V}\mathrm{M}\mathrm{P}}}_{\mathrm{i}}\mathrm{*}{\upbeta\:}+{\mathrm{p}}_{\mathrm{i}},\:$$

where $\:{y}_{{2}_{ij}}$ is the OSE phenotype for individual i in event j, $\:{\mathrm{O}\mathrm{S}\mathrm{I}\_\mathrm{E}\mathrm{B}\mathrm{V}\mathrm{M}\mathrm{P}}_{\mathrm{i}}$ is the EBV from masked phenotypes for OSI of individual i, $\:{\upbeta\:}$ is the regression coefficient of $\:{\mathrm{y}}_{2}^{}$on OSI EBVMPs, and the other effects are as previously described.

The accuracy formula described above was then applied using the newly obtained regression coefficient, the variance of the EBVMPs used as predictors, and the variance components from the validation model. Continuing the same example, the formula for across-trait accuracy for the $\:\mathrm{O}\mathrm{S}\mathrm{E}-{\mathrm{O}\mathrm{S}\mathrm{I}}_{\mathrm{E}\mathrm{B}\mathrm{V}\mathrm{M}\mathrm{P}}$ combination was:

15 $$\:{{\uprho\:}}_{\left({\:\mathrm{A}}_{\mathrm{O}\mathrm{S}\mathrm{E}},\:{\:\mathrm{E}\mathrm{B}\mathrm{V}\mathrm{M}\mathrm{P}}_{\mathrm{O}\mathrm{S}\mathrm{I}}\right)}=\frac{\sqrt{\frac{{\widehat{{\upbeta\:}}}^{2}{{\upsigma\:}}_{\mathrm{O}\mathrm{S}{\mathrm{I}}_{\mathrm{E}\mathrm{B}\mathrm{V}\mathrm{M}\mathrm{P}}}^{2}}{{\widehat{{\upbeta\:}}}^{2}{{\upsigma\:}}_{\mathrm{O}\mathrm{S}{\mathrm{I}}_{\mathrm{E}\mathrm{B}\mathrm{V}\mathrm{M}\mathrm{P}}}^{2}+\:\widehat{{{\upsigma\:}}_{{p}_{OSE}}^{2}}+\widehat{{{\upsigma\:}}_{{\mathrm{e}}_{\mathrm{O}\mathrm{S}\mathrm{E}}}^{2}}}}}{{\mathrm{h}}_{\mathrm{O}\mathrm{S}\mathrm{E}}},$$

where $\:{{\uprho\:}}_{({\:\mathrm{A}}_{\mathrm{O}\mathrm{S}\mathrm{E}},\:{\:\mathrm{E}\mathrm{B}\mathrm{V}\mathrm{M}\mathrm{P}}_{\mathrm{O}\mathrm{S}\mathrm{I}})}$ is the estimated correlation between EBVMPs for OSI and the true breeding values for OSE, $\:{\widehat{{\upbeta\:}}}^{2}{{\upsigma\:}}_{\mathrm{O}\mathrm{S}\mathrm{I}\_\mathrm{E}\mathrm{B}\mathrm{V}\mathrm{M}\mathrm{P}}^{2}$ represents the variance of OSI EBVMPs multiplied by the square of the regression coefficient from the between-trait validation model,$\:\:\widehat{{{\upsigma\:}}_{{\mathrm{p}}_{\mathrm{O}\mathrm{S}\mathrm{E}}}^{2}}$ is the estimated permanent environmental variance for OSE obtained from the original variance component model, $\:\widehat{{{\upsigma\:}}_{{\mathrm{e}}_{\mathrm{O}\mathrm{S}\mathrm{E}}}^{2}}$ is the residual variance for OSE (fixed to one), and $\:{\mathrm{h}}_{\mathrm{O}\mathrm{S}\mathrm{E}}$ is the square root of the estimated heritability for OSE in the liability scale.

A non-zero between-trait accuracy indicates that EBVMPs from the predictor trait capture genetic effects that also influence the target trait, providing evidence for a genetic correlation between the two traits. When EBVs are based on masked phenotypes, the relationship of within and between-trait cross-validation accuracies for the previous example is given by

16 $$\:{{\uprho\:}}_{\left(\:{\mathrm{A}}_{\mathrm{O}\mathrm{S}\mathrm{E},\:}\:{\mathrm{E}\mathrm{B}\mathrm{V}\mathrm{M}\mathrm{P}}_{\mathrm{O}\mathrm{S}\mathrm{I}}\right)}={{\uprho\:}}_{{\mathrm{E}\mathrm{B}\mathrm{V}\mathrm{M}\mathrm{P}}_{\mathrm{O}\mathrm{S}\mathrm{I}}}\:\:\mathrm{r}\left({\mathrm{A}}_{\mathrm{O}\mathrm{S}\mathrm{E}},\:{\mathrm{A}}_{\mathrm{O}\mathrm{S}\mathrm{I}}\right),$$

where $\:{{\uprho\:}}_{\left(\:{\mathrm{A}}_{\mathrm{O}\mathrm{S}\mathrm{E},\:}\:{\mathrm{E}\mathrm{B}\mathrm{V}\mathrm{M}\mathrm{P}}_{\mathrm{O}\mathrm{S}\mathrm{I}}\right)}$ is the between-trait accuracy, $\:{{\uprho\:}}_{{\mathrm{E}\mathrm{B}\mathrm{V}\mathrm{M}\mathrm{P}}_{\mathrm{O}\mathrm{S}\mathrm{I}}}$ is the within-trait accuracy for OSI, and $\:\mathrm{r}\left({\mathrm{A}}_{\mathrm{O}\mathrm{S}\mathrm{E}},\:{\mathrm{A}}_{\mathrm{O}\mathrm{S}\mathrm{I}}\right)$ is the genetic correlation between OSE and OSI. Therefore, we estimated the genetic correlation for the previous example as:

17 $$\:\mathrm{r}\left({\mathrm{A}}_{\mathrm{O}\mathrm{S}\mathrm{E}},\:{\mathrm{A}}_{\mathrm{O}\mathrm{S}\mathrm{I}}\right)=\:\frac{{{\uprho\:}}_{\left(\:{\mathrm{A}}_{\mathrm{O}\mathrm{S}\mathrm{E},\:}\:{\mathrm{E}\mathrm{B}\mathrm{V}\mathrm{M}\mathrm{P}}_{\mathrm{O}\mathrm{S}\mathrm{I}}\right)}}{{{\uprho\:}}_{{\mathrm{E}\mathrm{B}\mathrm{V}\mathrm{M}\mathrm{P}}_{\mathrm{O}\mathrm{S}\mathrm{I}}}},$$

A step-by-step derivation of this relationship is provided in Appendix B.

The genetic correlation formula was applied to all pairwise trait combinations. For each pair, correlations were estimated in both directions (e.g., $\:\mathrm{O}\mathrm{S}\mathrm{E}-{\mathrm{O}\mathrm{S}\mathrm{I}}_{\mathrm{E}\mathrm{B}\mathrm{V}\mathrm{M}\mathrm{P}}$ and $\:\mathrm{O}\mathrm{S}\mathrm{I}-{\mathrm{O}\mathrm{S}\mathrm{E}}_{\mathrm{E}\mathrm{B}\mathrm{V}\mathrm{M}\mathrm{P}}$), and their mean was taken as the final estimate of the genetic correlation between the two traits.

## Results

### Descriptive statistics

**Fig. 4:**
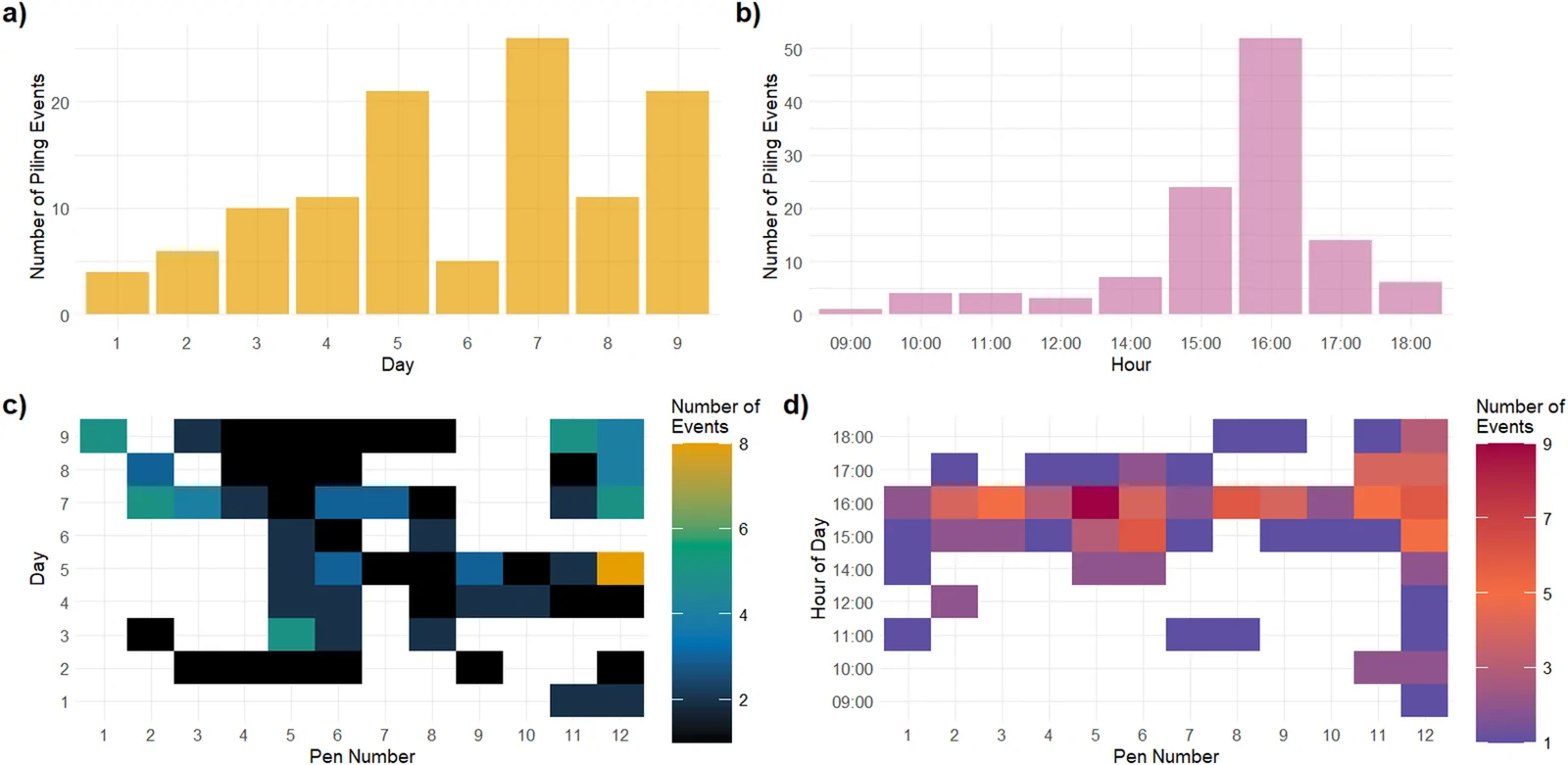
Piling event occurrence per day, hour and pen. (**a**) aggregated number of events in each day, (**b**) total number of events happening per aggregated hour of the day, (**c**) number of events happening for each pen-day combination, (**d**) number of events happening for each pen-hour combination

**Fig. 5:**
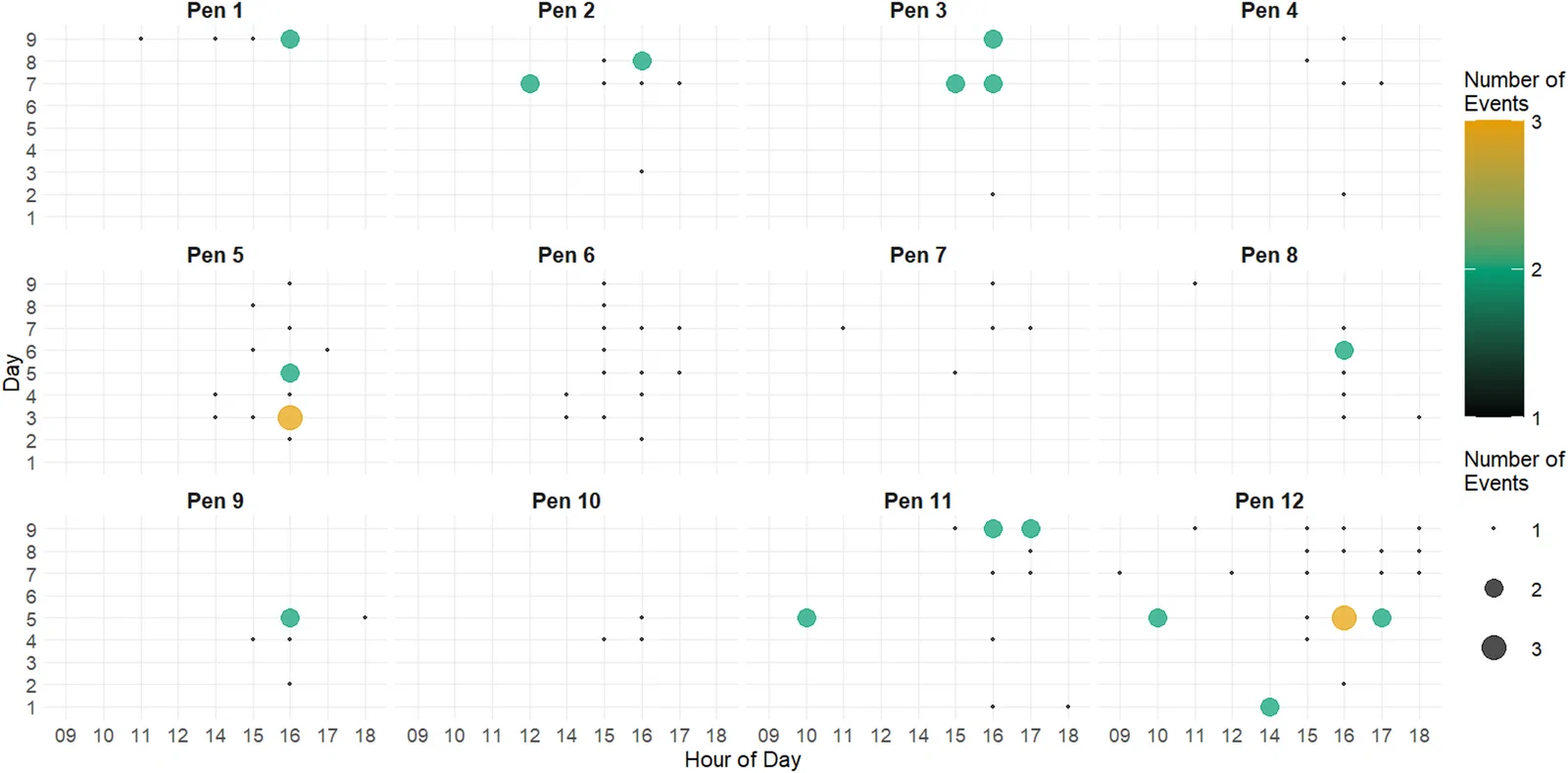
Piling event occurrence per each pen-day-hour combination

Piling events occurred in very heterogeneous patterns across pens, days, and hours (Figs. 4 and 5). Overall, Pen 12 showed the highest aggregated number of piling events (Figs. 4c and d and 5). However, this high activity was heavily concentrated on day 5, when multiple events occurred within the same hour (Fig. 5). Events in Pen 12 occurred predominantly in the late afternoon (Figs. 4d and 5).

In contrast, Pen 6 exhibited more consistent daily piling activity, showing at least one event on every observed day between 14:00 and 17:00. Within this period, only one event occurred per hour, with 15:00 being the most common hour (Figs. 4d and 5). Conversely, Pen 10 showed the least piling activity, with a total of only three events occurring between 15:00 and 16:00 on days 4 and 5 (Figs. 4c and 5).

Pens 1, 3, 4, 7, and 9 each showed approximately five events in total, but their temporal distributions varied. For instance, all events in Pen 1 were clustered on day 9, while the events in the other pens were spread across multiple days (Figs. 4c and 5). A peak of piling activity was observed at 16:00 across days (Fig. 4b and d), with a noticeable increase in total piling events across the facility occurring on days 5, 7, and 9 (Fig. 4a). Day 7 exhibited the largest single-day piling event count (Fig. 4a and c).

**Fig. 6:**
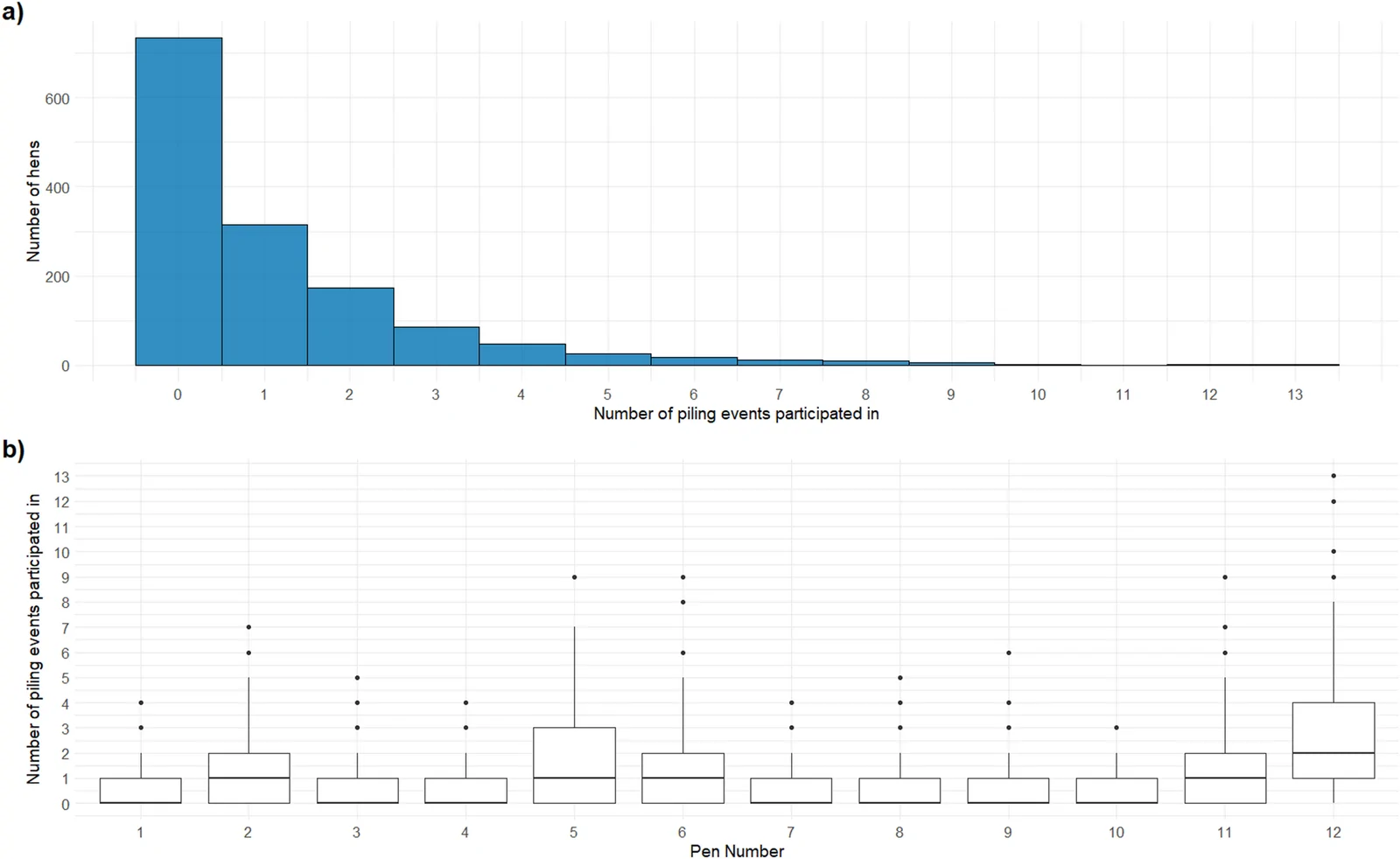
Distribution of piling participation among individual hens. (**a**) Histogram showing the number of piling events in which individual hens participated during the study period. (b) Distribution of the number of piling events participated in by individual hens within each pen, shown as boxplots

Differences in piling activity among pens were also reflected in the participation frequencies of individual hens (Fig. 6). Approximately 51% of hens did not participate in any piling event during the study period, while 34% participated in one or two piling events, 11% participated in three to five events, and 3% participated in six to ten events. Only two individuals (0.1%) participated in more than ten piling events, with the maximum participation count reaching 13 events. Pens 5, 6, 11 and 12 contained the individuals with the highest participation frequencies (Fig. 6b). Pen 12 additionally showed the highest median participation frequency (2 events), whereas median participation frequencies in the remaining pens ranged from 0 to 1 event. This suggests that the higher piling activity observed in Pen 12 was likely accompanied by more frequent participation across individuals within the pen.

Table 2 summarizes descriptive statistics for all three piling traits, together with the estimated raw variance for day and for pen obtained from separate models. For OSI (overall participation in piling), approximately half of the individuals participated in at least one piling event during the entire observation period. The lower prevalence for OSE (per-event piling participation) and the low mean for RSE (order of joining within each piling event) are both influenced by the annotation process, as only a subset of individuals per event was annotated as pile participants. Individuals who were present but not annotated were assigned a score of zero. This reduced the event-level prevalence for OSE and pulled the overall mean for RSE down.

Raw variances showed that pen effects were significant only for OSI; this was expected due to the individual-level average nature of the trait (ever/never participated in a pile across the whole observation period), and therefore, there was no data to estimate day-to-day variation. This suggested that piling was influenced more by temporal factors than by the housing pen.

**Table 2:** Descriptive Statistics of traits

Trait	N records (min, max)	Prevalence / Mean (SD)	Raw Day variance (CI99)	Raw Pen variance (CI99)
OSI	1,411 (0,1)	0.485 (0.50)	-	0.108 (0.035, 0.426)
OSE	13,585 (0,1)	0.118 (0.32)	0.012 (0.001, 0.074)	0.001 (0.00, 0.001)
RSE	13,585 (0,1)	0.064 (0.20)	9.21 × 10^− 5^ (0.00, 0.5 × 10^− 3^)	4.83 × 10^− 6^ (0.00, 0.1 × 10^− 3^)

OSI: one score per individual, OSE: one score per event, RSE: rank score per event, N records: total number of scores available for the trait, min: minimum score value, max: maximum score value, SD: standard deviation, Variance components and their 99% confidence intervals (CI99) were estimated from separate models with only an intercept and a random effect for Day or for Pen

### Genetic analysis

All three traits showed significant genetic variance (Table 3), which indicates that genetic differences among individuals contribute to variation in OSI, OSE, and RSE. The variance of the permanent effect was substantial for both OSE and RSE. For RSE, it was more than double the genetic variance, whereas for OSE, the permanent environmental and genetic variances were practically the same. This suggested that non-genetic individual-specific factors were larger for RSE.

Despite low point estimates, all heritabilities were highly significant (*p* < 0.01), as indicated by CI99, calculated as the estimate ± 2.576 × SE. On the liability scale, the CI99 ranged from 0.039 to 0.301 for OSI and from 0.055 to 0.225 for OSE. On the observed scale, intervals were 0.061–0.159 for OSI, 0.019–0.081 for OSE, and 0.008–0.091 for RSE. These results confirm that genetic variance significantly contributes to the variation in all three piling *traits.*

**Table 3:** Heritability and genetic parameters across piling phenotypes

Trait	$\:{{\upsigma\:}}_{\mathrm{a}}^{2}$ (± SE)	$\:{{\upsigma\:}}_{\mathrm{p}}^{2}$ (± SE)	$\:{{\upsigma\:}}_{\mathrm{e}}^{2}$ (± SE)	$\:{\mathrm{h}}_{\mathrm{l}\mathrm{i}\mathrm{a}\mathrm{b}}^{2}$ (± SE)	$\:{\mathrm{h}}_{\mathrm{o}\mathrm{b}\mathrm{s}}^{2}$ (± SE)	Rep
OSI	0.203 (± 0.074)	-	1 (fixed)	0.17 (± 0.051)	0.11 (± 0.033)	-
OSE	0.195 (± 0.053)	0.213 (± 0.034)	1 (fixed)	0.14 (± 0.033)	0.05 (± 0.012)	0.30
RSE	0.002 (± 0.0007)	0.005 (± 0.0005)	0.035 (± 0.0004)	-	0.05 (± 0.016)	0.16

OSI: one score per individual, OSE: one score per event, RSE: rank score per event, $\:{{\upsigma\:}}_{\mathrm{a}}^{2}$: genetic variance, $\:{{\upsigma\:}}_{\mathrm{p}}^{2}$: permanent environmental variance, $\:{{\upsigma\:}}_{\mathrm{e}}^{2}$: residual variance, $\:{\mathrm{h}}_{\mathrm{o}\mathrm{b}\mathrm{s}}^{2}$: heritability on the observed scale, $\:{\mathrm{h}}_{\mathrm{l}\mathrm{i}\mathrm{a}\mathrm{b}}^{2}$: heritability on liability scale, Rep: repeatability = $\:{({\upsigma\:}}_{\mathrm{a}}^{2}+\:{{\upsigma\:}}_{\mathrm{p}}^{2})\:/(\:{{\upsigma\:}}_{\mathrm{a}}^{2}+{{\upsigma\:}}_{\mathrm{p}}^{2}+\:{{\upsigma\:}}_{\mathrm{e}}^{2})$, SE: standard error

### Simulation study

Results of the simulation study are presented in Table 4. The continuous model estimated the genetic variance and heritability almost exactly, with mean estimates of both parameters differing by less than 1% from the true values. The complete OSE model produced estimates that were nearly identical to those obtained with the simplified threshold model, despite allowing estimation of a permanent environmental effect that was not simulated. Both the simplified threshold model and the complete OSE model slightly underestimated the genetic variance and heritability by approximately 10–12%, whereas the OSI model slightly overestimated the genetic variance by approximately 14%, and heritability by approximately 10%. Nevertheless, the deviations from the true values were modest, suggesting limited bias.

**Table 4:** Mean estimated variance components and heritabilities obtained from 100 simulation replicates

Model	Mean $\:{{\upsigma\:}}_{\mathrm{a}}^{2}$ ($\:\stackrel{-}{\mathrm{S}\mathrm{E}}$)	Mean $\:{{\upsigma\:}}_{\mathrm{p}}^{2}$ ($\:\stackrel{-}{\mathrm{S}\mathrm{E}}$)	Mean $\:{{\upsigma\:}}_{\mathrm{e}}^{2}$ ($\:\stackrel{-}{\mathrm{S}\mathrm{E}}$)	Mean h² ($\:\stackrel{-}{\mathrm{S}\mathrm{D}}$)	Number of simulations
True	0.25	–	1	0.20	–
Continuous	0.248 (0.017)	–	1 (0.012)	0.198 (0.012)	100
Simple binary	0.215 (0.022)	–	–	0.177 (0.016)	100
OSE	0.219 (0.024)	0.002 (0.016)	–	0.179 (0.016)	100
OSI	0.285 (0.088)	–	–	0.220 (0.032)	100

$\:{{\upsigma\:}}_{\mathrm{a}}^{2}$: genetic variance, $\:{{\upsigma\:}}_{\mathrm{p}}^{2}$: permanent environmental variance, $\:{{\upsigma\:}}_{\mathrm{e}}^{2}$: residual variance, $\:{\mathrm{h}}^{2}$= heritability, $\:\stackrel{-}{\mathrm{S}\mathrm{E}}$: mean standard error of variance estimation across simulations; $\:\stackrel{-}{\mathrm{S}\mathrm{D}}$: mean standard deviation of heritability across simulation. True: simulated values of $\:{{\upsigma\:}}_{\mathrm{a}}^{2}$, $\:{{\upsigma\:}}_{\mathrm{e}}^{2}$, and $\:{\mathrm{h}}^{2}$. Continuous: animal model fitted to the simulated continuous phenotype. Simple binary: simplified threshold model fitted to the simulated OSE phenotype. OSE: complete threshold model fitted to the simulated OSE phenotype. OSI: threshold model fitted to the simulated OSI phenotype

### Predicted response to selection

**Fig. 7:**
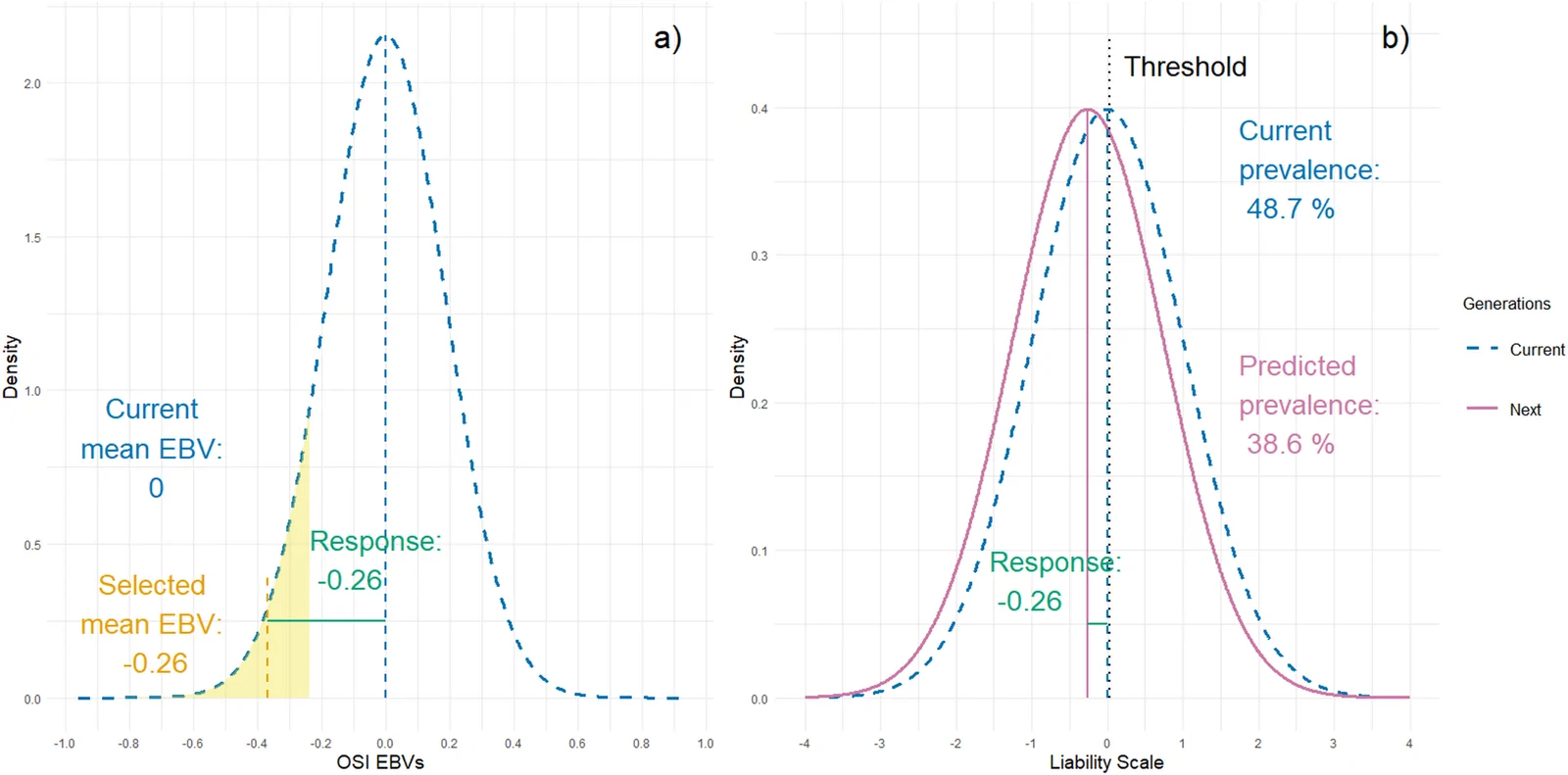
OSI predicted response to selection. (**a**) OSI EBV distribution showing selection differential when selecting the 10% individuals with the lowest EBV. (**b**) Change in prevalence, on the liability scale, after selection

**Fig. 8:**
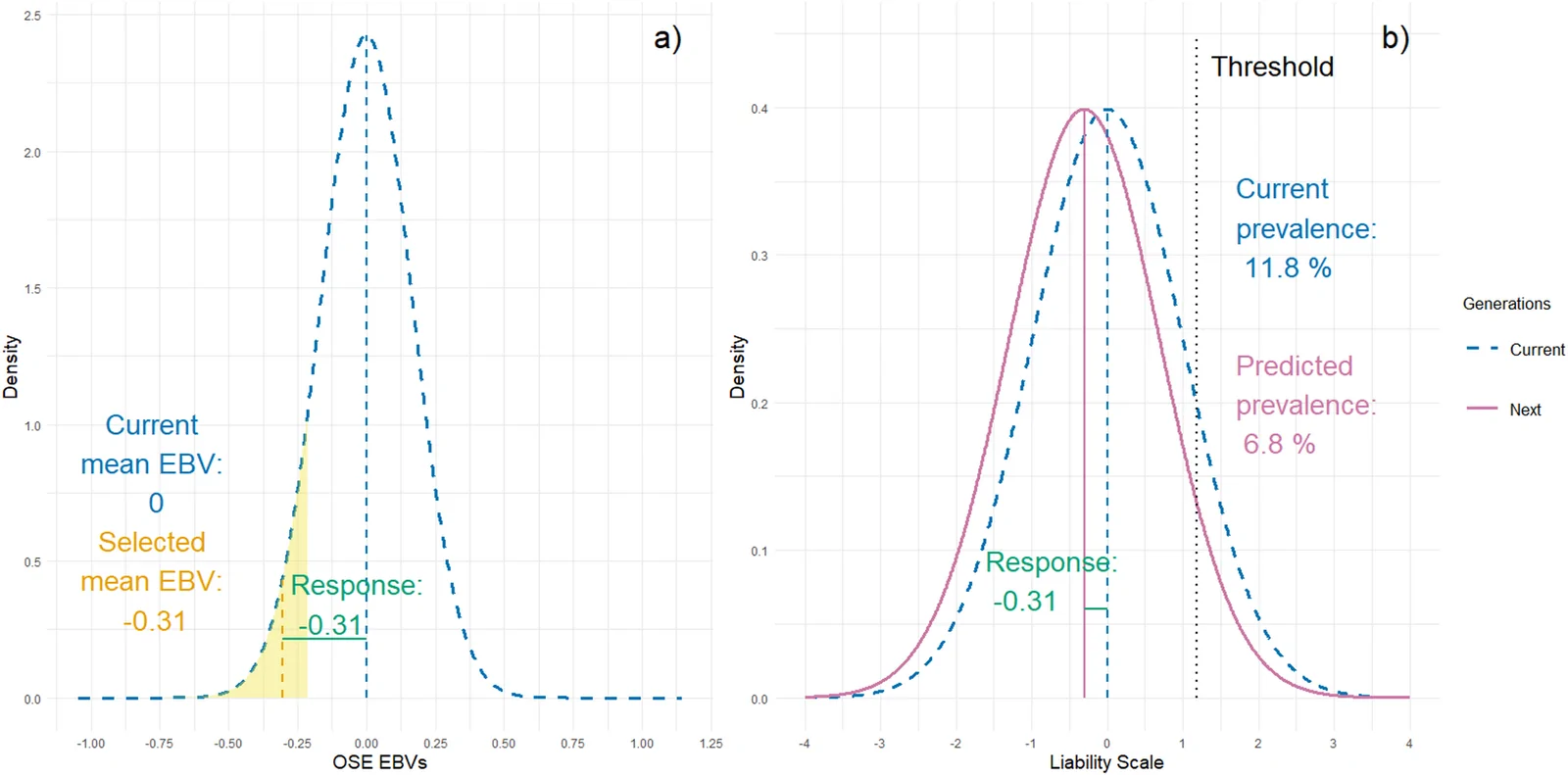
OSE predicted response to selection. (**a**) OSE EBV distribution showing selection differential when selecting the 10% individuals with the lowest EBV. (**b**) Change in prevalence, on the liability scale, after selection

**Fig. 9:**
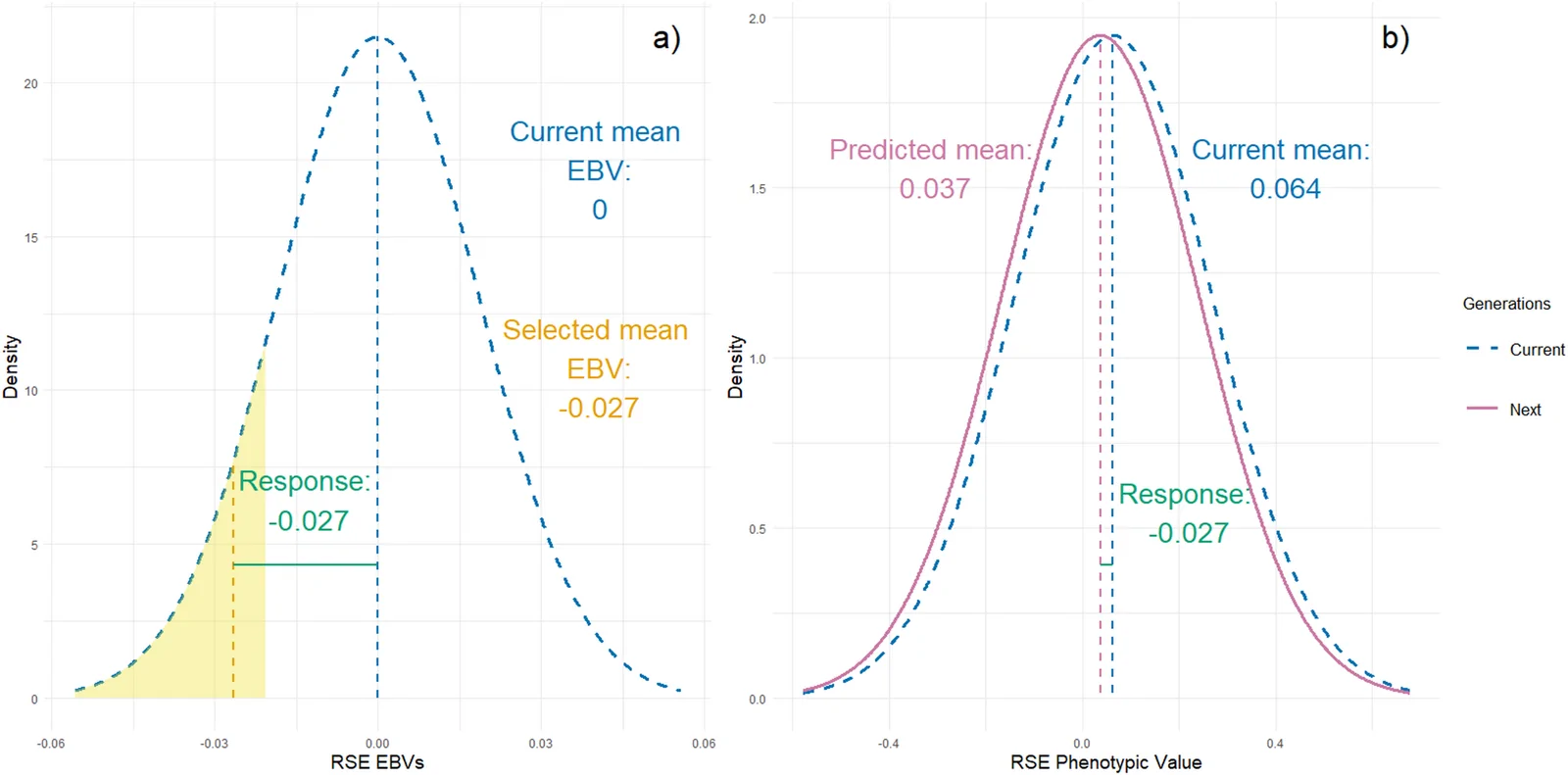
RSE predicted response to selection. (**a**) RSE EBV distribution showing selection differential when selecting the 10% individuals with the lowest EBV. (**b**) Change in phenotypic mean, after selection

The predicted response to selection for the lowest 10% of EBVs is shown in Figs. 7 and 8, and 9. For OSI, selection is predicted to reduce the mean EBV from 0 to -0.26 (Fig. 7a), decreasing the prevalence from 48.7% to 38.6% (Fig. 7b). This decrease represents a predicted absolute reduction of 10.1% and a relative reduction of 21%. For OSE, the mean EBV is predicted to decrease to -0.31 (Fig. 8a), reducing prevalence from 11.8% to 6.8% (Fig. 8b), a predicted relative reduction of 42%. For RSE, a selection differential of -0.027 is predicted (Fig. 9a), resulting in a mean phenotypic value reduction from approximately 0.06 to 0.04 (Fig. 9b), a predicted relative reduction of 33%. In all cases, these predicted shifts suggest that selection can be effective for meaningful reductions in the mean value of the traits.

### Cross validation and predictive accuracy

Cross-validation accuracies for individuals with masked phenotypes were moderate across all traits (Table 5). For all traits, the model-based within-trait accuracies were substantially higher than the corresponding cross-validation estimates, as is often observed with genomic prediction.

For the within-trait validations, the regression coefficients of phenotypes on the EBVMPs were all significantly different from zero, confirming that the predictions captured a true genetic signal rather than random noise. To assess the dispersion of the EBVMPs in the within-trait cross-validation, we examined whether these coefficients were significantly different from one. The CI99 (calculated as $\:\widehat{{\upbeta\:}}$ ± 2.576 × SE) for all traits included one (OSI: 0.850–2.669; OSE: 0.567–1.392; RSE: 0.425–1.455). Hence, even though the cross-validation accuracies were smaller than the reported accuracies, we found no evidence of over- or underdispersion of EBVs. However, the considerable width of these intervals reflects substantial uncertainty about the precise scaling of the predictions.

Accuracies across all trait combinations were similar to the within-trait accuracies, indicating that the genetic information captured by one measure of piling behaviour largely overlaps with that captured by the others. For instance, EBVMPs derived from OSE predicted OSI with an accuracy slightly higher than the within-trait value for OSI (0.49 vs. 0.44; Table 5). Across all trait combinations, accuracies were highest for OSI as target trait while using phenotypes for OSE as information source. Thus, breeding values can be predicted most accurately for OSI, while OSE records provided the most information.

As expected, the between-trait accuracies were not symmetrical. For example, the accuracy of predicting OSI using OSE EBVMPs was higher than that of predicting OSE using OSI EBVMPs. A similar asymmetry was observed when comparing RSE–OSI_EBVMP_ and RSE–OSE_EBVMP_ combinations to their reverse combinations. These differences likely reflect the unequal heritabilities of the traits.

In the between-trait analyses, the large differences in regression coefficient arise due to differences in the measurement scales (e.g., observed vs. liability scale). Hence, our main interest was in the statistical significance of the estimated regression coefficients, not in their precise value. All between-trait regression coefficients were significantly different from zero (*p* < 0.01), confirming that EBVMPs from one trait captured meaningful predictive information for another.

**Table 5:** Within-trait and between-trait accuracy

Target trait	EBVMP source	$\:\sigma_{\mathrm{EBVMP}}^{\mathrm{2}}$	$\:\widehat{{\upbeta\:}}$ (SE)	CV accuracy	Reported accuracy
Within-trait					
OSI	OSI	0.0109	1.76 (0.33)	0.44	0.63
OSE	OSE	0.0196	0.98 (0.16)	0.32	0.67
RSE	RSE	0.0002	0.94 (0.20)	0.27	0.65
Between-trait					
OSI	OSE	0.0196	1.46 (0.25)	0.49	–
OSI	RSE	0.0002	13.16 (2.60)	0.42	–
OSE	OSI	0.0109	1.25 (0.22)	0.30	–
OSE	RSE	0.0002	9.21 (1.68)	0.28	–
RSE	OSI	0.0109	0.13 (0.03)	0.29	–
RSE	OSE	0.0196	0.10 (0.02)	0.31	–

OSI: one score per individual, OSE: one score per event, RSE: rank score per event; EBVMP source: Trait of origin for EBVMPs; $\:{{\upsigma\:}}_{\mathrm{E}\mathrm{B}\mathrm{V}\mathrm{M}\mathrm{P}}^{2}$: variance of EBVMPs; $\:\widehat{{\upbeta\:}}$: regression coefficient of phenotypes on EBVMPs, CV Accuracy: cross-validation accuracy; Reported accuracy: model-based accuracy

The estimated genetic correlations between all pairs of piling traits were near one (Table 6). These values exceed the theoretical maximum of one due to sampling variation in the accuracies used for the calculation. Nevertheless, these estimates suggest that all the traits share an extensively overlapping genetic background.

**Table 6:** Mean estimated genetic correlation between traits

Trait 1	Trait 2	Mean genetic correlation estimate^1^
OSE	OSI	1.11
OSE	RSE	1.01
OSI	RSE	1.10

OSI: one score per individual, OSE: one score per event, RSE: rank score per event. ^1^ Genetic correlations were estimated using cross-validation. For this reason, the estimates were not forced to be within the [-1, 1] parameter space

## Discussion

This study provides the first quantitative genetic analysis of piling behaviour in laying hens, focusing on individual-level behaviour. We demonstrated that a hen’s tendency to participate in piling events is heritable. While the occurrence of piling events varied across pens and days, the individual’s behavioural response to them showed a clear genetic basis. These findings establish that piling is a heritable trait and lay the groundwork for its mitigation through selection if desired.

Our findings show that piling behaviour was expressed in heterogeneous and non-random patterns. Event occurrence was heavily clustered within a subset of pens and consistently peaked during the late afternoon, with an increase around 16:00 h (Figs. 4 and 5). This temporal pattern aligns with known diurnal rhythms in laying hens. The late afternoon is typically characterized by increased general activity, foraging, and pre-roosting social interactions (e.g [27]), . Piling behaviour appears to be expressed within this behavioural context, suggesting it may be facilitated by a general increase in mobility and social motivation during this period.

Participation in piling events was concentrated in a subset of individuals (Fig. 6a), indicating consistent differences among hens in their propensity to join piling events. Interestingly, pens containing the most frequent participants also tended to exhibit a higher median of participation (Fig. 6b), which suggested that the higher piling activity within these pens was not restricted to a few highly participating hens. One possible explanation is that the probability of joining a piling event may be influenced both by an individual’s own propensity to pile and by the behaviour of its pen mates. Such social effects are common in behavioural traits and may arise through, for example, indirect genetic effects (e.g [28, 29]), . Although the present study focused on simplified individual-level phenotypes, future studies may benefit from including social genetic effects in piling behaviour analysis.

The stocking density of approximately 13 birds/m² used in this study was higher than the maximum of 9 birds/m² permitted for commercial laying hen housing within the European Union [30]. This higher stocking density was intended to promote social interactions among birds. Stocking density has been shown to influence some social behaviours in laying hens, including feather pecking and aggression [31]. It is therefore possible that stocking density contributed to the frequency of piling observed in the present study. However, the causes of recurrent piling events remain poorly understood [4] and the relationship between stocking density and piling behaviour has not been clearly established.

For the analysis of the traits, we modelled pen and event number as fixed effects. On the one hand, this is conservative because it ensures that the genetic variance is estimated only from differences among individuals housed within the same pen, thereby preventing an effect of potential confounding between pen environment and genetic effects on the genetic variance estimate [32]. Although previous genomic relatedness analyses confirmed that individuals were effectively randomized across pens [33], we preferred to exclude between-pen information from the genetic variance estimates to be as conservative as possible. Moreover, the fixed effects accounted for differences in the trait mean between pens; for RSE, such differences may reflect different number of annotated piling participants. Together, the use of pen and event number as fixed effects, the lack of confounding between pens and genetic effects, and the results from the cross-validation strongly suggest that the genetic variance estimated for the traits reflects a genuine biological signal rather than a modelling artifact.

On the other hand, treating pen and event number as fixed effects is anti-conservative, because it removes differences in the response variable due to pen effects from the variance of the model (i.e., from the “noise”). This may result in underestimation of standard errors, P-values that are too small, and false significance of model terms.

Importantly, the choice between fixed and random modelling had a negligible impact on breeding values obtained under both specifications, which were nearly identical with a correlation of ~ 0.99 (data not shown). Also, the estimates of genetic variance were robust to the effects being fitted either as fixed or random because their values were very similar (0.528 vs. 0.459 for OSI, 0.195 vs. 0.187 for OSE, and 0.0022 vs. 0.0021 for RSE). This consistency, together with the small variance explained by Day or Pen in preliminary models (Table 2), and the large number of observations per day and per pen, indicates that the choice of fixed vs. random effects is not critical here.

The genetic parameters and EBVs in this study were estimated using a GRM constructed from the three-way crossbred hens. In crossbred populations, linkage disequilibrium (LD) between SNP markers and causal loci may differ among the contributing lines. Thus, a marker that is in strong LD with a causal locus in one line may be in weaker LD, or no LD with that locus in another line [34]. However, genomic relationships between animals from different lines generally show little variation of relatedness compared with animals from the same line [35]. Therefore, most of the variation in genomic relationships used to estimate genetic effects comes from within line comparisons. Consequently, differences in LD among the contributing lines are expected to have a limited effect on the genetic covariance captured by the GRM.

The models assumed additive effects and did not explicitly estimate non-additive genetic effects. If additive and non-additive effects are independent, non-additive variation that is omitted from the model is not attributed to the additive variance [36, 37]. However, if these effects are not independent, some non-additive variation may be captured by the additive component, potentially affecting the estimated additive genetic variance and heritability [36]. Therefore, omission of non-additive effects does not necessarily result in overestimation of the additive genetic variance, although some contribution of non-additive effects to the estimates cannot be excluded.

The differences in heritability between OSI, OSE and RSE (Table 3) must be interpreted in the context of their measurement. The higher heritability for OSI may partly reflect its nature as a kind of individual-level average. By condensing an animal’s piling behaviour across the entire observation period into a single value, OSI to some degree averages out the event-specific environmental noise that is captured in the OSE model, potentially resulting in a higher heritability estimate. Notably, the estimated genetic variance for OSI was larger than that for OSE. This could suggest that the overall propensity to pile (OSI) is influenced more strongly by genetic factors than the probability of joining a pile in any single event (OSE). However, the simulation study suggests that part of this difference may also arise from the phenotype definitions and the models used for their analysis. The complete model for simulated OSE phenotypes tended to slightly underestimate genetic variance and heritability, whereas the model for simulated OSI phenotypes slightly overestimated these parameters (Table 4). Therefore, the larger genetic variance estimated for OSI in the present study may reflect both biological and methodological differences.

Furthermore, to limit the required labour investments, we annotated a maximum of 20 participants per event. This protocol means that for large piles, the phenotypes for that trait are partially inaccurate; an individual may have participated but was recorded as a non-participant (phenotype = 0). This annotation error likely adds noise, which could bias heritability estimates downward. An automated, full-pen tracking system that can reliably identify all participants in every pile could eliminate this issue and provide less noisy data for genetic analysis. Nevertheless, this type of automatic individual-level phenotyping is yet to be fully developed. Recent progress in computer-vision methods, including the group-level pile detection used in our study and in other efforts (e.g [38, 39].), demonstrates that automated detection of pile events is feasible. However, individual-level tracking within dense piles remains technically challenging due to occlusion, where hens are hidden from the view of the camera (e.g [40]).

The potential response to selection for all traits, with relative reductions ranging from 21% to 42% (Figs. 7, 8 and 9), suggests that genetic selection could help to reduce piling. Because we found no evidence of inflation of EBV, as confidence intervals for $\:\widehat{{\upbeta\:}}$ included the value of one, we did not down-scale the EBV in the calculation of response to selection. However, as with any other binary trait, long-term selection faces challenges: as prevalence declines, observed‑scale heritability decreases, reducing EBV accuracy (e.g [41]), . One approach to address this challenge could be to house hens in environments that facilitate piling expression, such as aviary pens that were used in the current study.

Reducing piling through genetic selection could improve animal welfare by decreasing the occurrence of piling events and their associated consequences, for example, injuries and mortality due to smothering. Furthermore, if hens genetically predisposed to participate in piling events are overrepresented within a group, a greater number of individuals may join a pile once it has formed, potentially increasing pile size and the severity of its welfare consequences. Consequently, selection against the propensity to participate in piling behaviour could reduce not only the occurrence of piling events but also their severity. However, future research should evaluate genetic correlations of piling with other welfare and production traits to avoid unintended trade-offs.

The cross-validation confirmed that the estimated breeding values capture a real genetic signal, as all within-trait regression coefficients were significantly different from zero (Table 5). This indicates that the EBVMPs are not merely random noise but reflect underlying genetic differences for piling propensity. The considerable width of the CI99 for the within-trait regression coefficients complicates the precise scaling of the predictions; however, this does not affect their accuracy.

A methodological convenience of our validation models is that they insert the regression on EBVMPs within the GLMM framework, allowing accuracy to be calculated directly on the liability scale. Since the model’s internal response variable for OSI and OSE operates on this scale, the resulting accuracy reflects the correlation between EBVMPs and underlying genetic merit without the need for post-hoc transformations.

Based on our trait definitions, we expected high genetic correlations between traits, which is supported by the between-trait predictive accuracies in Table 5. EBVMPs from one trait predicted phenotypic variation in another with accuracies approaching within-trait values, indicating a strong genetic correlation. The observed asymmetries in these accuracies were expected. While the genetic correlation is symmetrical, the predictive accuracy is not; the accuracy of predicting trait Y using EBVMPs from trait X is different from predicting X using Y’s EBVMPs. This occurs because predictive accuracy depends on the accuracy of the predictor EBVMPs (see Methods).

Because bivariate models failed to converge, we estimated genetic correlations indirectly by averaging the ratio of between‑trait to within‑trait predictive accuracy for each trait pair in both directions (see Methods). All resulting estimates slightly exceeded 1.0 (Table 6), which is due to sampling variance. Nevertheless, they suggest that the true genetic correlations are very high.

Interestingly, the strongest between-trait prediction accuracy was obtained when OSE EBVMPs were used to predict OSI, with an accuracy that even exceeded OSI’s within-trait value (0.49 vs. 0.44; Table 5). This pattern indicates that the event-level information captured by OSE is informative for estimating the underlying, overall tendency to pile. In practical terms, this suggests that OSI could serve as the target trait for genetic evaluation, while OSE phenotypes could be used to enhance the accuracy of its prediction.

## Conclusion

This study shows that propensity to pile in laying hens is a heritable trait. We estimated significant genetic variance for all piling traits. Our analysis further revealed that the most accurate genomic predictions are achieved by modelling the overall predisposition to pile (OSI) using event-level participation data (OSE). These results establish a genetic basis for piling behaviour and its possible reduction through genomic selection. Future research should investigate the role of social genetic effects in piling behaviour and assess its correlation with welfare and production traits to prevent unwanted trade-offs during selection.

## Supplementary Information

Below is the link to the electronic supplementary material.

Supplementary Material 1

Supplementary Material 2

## Data Availability

The data is available upon request.

## References

[1] Majewski E, Potori N, Sulewski P, Wąs A, Mórawska M, Gębska M, et al. End of the cage age? A study on the impacts of the transition from cages on the EU laying hen sector. Agriculture. 2024;14:111.

[2] Lay DC, Fulton RM, Hester PY, Karcher DM, Kjaer JB, Mench JA, et al. Hen welfare in different housing systems. Poult Sci. 2011;90:278–94. 10.3382/ps.2010-00962 21177469

[3] Campbell DLM, Makagon MM, Swanson JC, Siegford JM. Litter use by laying hens in a commercial aviary: dust bathing and piling. Poult Sci. 2016;95:164–75. 10.3382/ps/pev183 26354762

[4] Gray H, Davies R, Bright A, Rayner A, Asher L. Why do hens pile? Hypothesizing the causes and consequences. Front Vet Sci. 2020;7:616836. 10.3389/fvets.2020.616836 PMC7758342 33363246

[5] Mazocco CC, de Castro Júnior SL, Silveira RMF, Poletto R, da Silva IJO. Laying hens: why smothering and not surviving?—a literature review. Anim (Basel). 2024;14:1518. 10.3390/ani14111518 PMC11171085 38891565

[6] Regmi P. Piling and smothering behaviors in commercial laying hens. Poultry Extension Collaborative. 2023. https://en.engormix.com/poultry-industry/behavioural-diseases-poultry/piling-smothering-behaviors-commercial_a52189/. Accessed 28 Aug 2026.

[7] Armstrong D, Asher L, Rayner A, Ngidda H, Sharma B, Gray H. The effect of piling behavior on the production and mortality of free-range laying hens. Poult Sci. 2023;102:102989. 10.1016/j.psj.2023.102989 PMC10465951 37604022

[8] Rentsch AK, Harlander A, Niel L, Siegford JM, Widowski TM. Raising laying hens: housing complexity and genetic strain affect startle reflex amplitude and behavioural response to fear-inducing stimuli. R Soc Open Sci. 2024;11:231075. 10.1098/rsos.231075 PMC10951723 38511084

[9] van Putten A, Giersberg MF, Rodenburg TB. Tracking laying hens with ArUco marker backpacks. Smart Agric Technol. 2025;10:100703.

[10] Alindekon S, Deutsch J, Rodenburg TB, Langbein J, Puppe B, Louton H. Tag ‘n’ Track: tackling the validation challenge in animal behaviour studies through automated referencing with ArUco markers. Comput Electron Agric. 2025;229:109812.

[11] Shilkrot R, Escriva DM. Mastering OpenCV 4: a comprehensive guide to building computer vision and image processing applications with C++. Packt Publishing Ltd; 2018.

[12] Van Rossum G, Drake FL. Python 3 reference manual. Scotts Valley, CA: CreateSpace; 2009.

[13] Posit team. RStudio: integrated development environment for R. Boston, MA: Posit Software, PBC; 2023.

[14] Purcell S, Neale B, Todd-Brown K, Thomas L, Ferreira MAR, Bender D, et al. PLINK: a tool set for whole-genome association and population-based linkage analyses. Am J Hum Genet. 2007;81:559–75. 10.1086/519795 PMC1950838 17701901

[15] VanRaden PM. Efficient methods to compute genomic predictions. J Dairy Sci. 2008;91:4414–23. 10.3168/jds.2007-0980 18946147

[16] Calus MPL, Vandenplas J. Calc_grm—a program to compute pedigree, genomic, and combined relationship matrices. ABGC, Wageningen UR Livestock Research;; 2016.

[17] Gilmour AR, Gogel BJ, Cullis BR, Welham SJ, Thompson R. ASReml user guide release 4.2 structural specification. Hemel Hempstead: VSN International Ltd; 2015.

[18] Robertson A. Appendix. In: Dempster ER, Lerner IM. Heritability of threshold characters. Genetics. 1950;35:212 – 36. 10.1093/genetics/35.2.212 PMC1209482 17247344

[19] Falconer DS, Mackay TFC. Introduction to quantitative genetics. 4th ed. Harlow: Addison Wesley Longman; 1996.

[20] Mrode RA. Linear models for the prediction of animal breeding values. 3rd ed. Wallingford: CABI; 2014.

[21] Alexandre PA, Li Y, Hine BC, Duff CJ, Ingham AB, Porto-Neto LR, et al. Bias, dispersion, and accuracy of genomic predictions for feedlot and carcase traits in Australian Angus steers. Genet Sel Evol. 2021;53:77. 10.1186/s12711-021-00673-8 PMC8474816 34565347

[22] David I, Ricard A, Huynh-Tran V-H, Dekkers JCM, Gilbert H. Quality of breeding value predictions from longitudinal analyses, with application to residual feed intake in pigs. Genet Sel Evol. 2022;54:32. 10.1186/s12711-022-00722-w PMC9103455 35562648

[23] Legarra A, Robert-Granié C, Manfredi E, Elsen J-M. Performance of genomic selection in mice. Genetics. 2008;180:611–8. 10.1534/genetics.108.088575 PMC2535710 18757934

[24] Matika O, Tarsani E, McIntosh K, Kebede FG, Talenti A, Rae AM, et al. Genomic selection accuracy and bias using imputed genotypes on growth, welfare and fitness traits in two Pekin duck lines. BMC Genomics. 2026;27:520. 10.1186/s12864-026-12855-z PMC13235070 42021140

[25] Maier RM, Zhu Z, Lee SH, Trzaskowski M, Ruderfer DM, Stahl EA, et al. Improving genetic prediction by leveraging genetic correlations among human diseases and traits. Nat Commun. 2018;9:989. 10.1038/s41467-017-02769-6 PMC5841449 29515099

[26] Montesinos-López A, Runcie DE, Ibba MI, Pérez-Rodríguez P, Montesinos-López OA, Crespo LA, et al. Multi-trait genomic-enabled prediction enhances accuracy in multi-year wheat breeding trials. G3 (Bethesda). 2021;11:jkab270. 10.1093/g3journal/jkab270 PMC8496321 34568924

[27] Campbell DLM, Makagon MM, Swanson JC, Siegford JM. Laying hen movement in a commercial aviary: enclosure to floor and back again. Poult Sci. 2016;95:176–87. 10.3382/ps/pev186 26195806

[28] Brinker T, Bijma P, Visscher J, Rodenburg TB, Ellen ED. Plumage condition in laying hens: genetic parameters for direct and indirect effects in two purebred layer lines. Genet Sel Evol. 2014;46:33. 10.1186/1297-9686-46-33 PMC4073196 24885199

[29] Worku Alemu S, Bijma P, Møller SH, Janss L, Berg P. Indirect genetic effects contribute substantially to heritable variation in aggression-related traits in group-housed mink (Neovison vison). Genet Sel Evol. 2014;46:30. 10.1186/1297-9686-46-30 PMC4046851 24884874

[30] Council of the European Union. Council Directive 1999/74/EC of 19 July 1999 laying down minimum standards for the protection of laying hens. Off J Eur Communities. 1999;L 203:53–7. https://eur-lex.europa.eu/legal-content/EN/TXT/?uri=CELEX:31999L0074. Accessed 18 Jun 2026.

[31] Nicol CJ, Gregory NG, Knowles TG, Parkman ID, Wilkins LJ. Differential effects of increased stocking density, mediated by increased flock size, on feather pecking and aggression in laying hens. Appl Anim Behav Sci. 1999;65:137–52.

[32] Patterson HD, Thompson R. Recovery of inter-block information when block sizes are unequal. Biometrika. 1971;58:545–54.

[33] Osorio-Gallardo T, van Putten A, Janssen D, Schrauf MF, Giersberg MF, Rodenburg TB, et al. From pixels to breeding values: genetic analysis of detection and movement traits in laying hens using automated tracking data of ArUco-marked birds. Genet Sel Evol. 2026;58:28. 10.1186/s12711-026-01043-y PMC13217794 41963780

[34] de Roos APW, Hayes BJ, Spelman RJ, Goddard ME. Linkage disequilibrium and persistence of phase in Holstein-Friesian, Jersey and Angus cattle. Genetics. 2008;179:1503–12. 10.1534/genetics.107.084301 PMC2475750 18622038

[35] Wilmot H, Niehoff T, Soyeurt H, Gengler N, Calus MPL. The use of a genomic relationship matrix for breed assignment of cattle breeds: comparison and combination with a machine learning method. J Anim Sci. 2023;101:skad172. 10.1093/jas/skad172 PMC10276639 37220912

[36] Muñoz PR, Resende MFR Jr, Gezan SA, Resende MDV, de los Campos G, Kirst M, et al. Unraveling additive from nonadditive effects using genomic relationship matrices. Genetics. 2014;198:1759–68. 10.1534/genetics.114.171322 PMC4256785 25324160

[37] Vitezica ZG, Varona L, Legarra A. On the additive and dominant variance and covariance of individuals within the genomic selection scope. Genetics. 2013;195:1223–30. 10.1534/genetics.113.155176 PMC3832268 24121775

[38] Bist RB, Subedi S, Yang X, Chai L. Effective strategies for mitigating feather pecking and cannibalism in cage-free W-36 pullets. Poultry. 2023;2:281–91.

[39] Jensen DB, Toscano M, van der Heide E, Grønvig M, Hakansson F. Comparison of strategies for automatic video-based detection of piling behaviour in laying hens. Smart Agric Technol. 2025;10:100745.

[40] Okinda C, Nyalala I, Korohou T, Okinda C, Wang J, Achieng T, et al. A review on computer vision systems in monitoring of poultry: a welfare perspective. Artif Intell Agric. 2020;4:184–208.

[41] Ellen ED, Ducrocq V, Ducro BJ, Veerkamp RF, Bijma P. Genetic parameters for social effects on survival in cannibalistic layers: combining survival analysis and a linear animal model. Genet Sel Evol. 2010;42:27. 10.1186/1297-9686-42-27 PMC2912826 20609233

