# Supplementary material for: Genetic parameters and genomic prediction accuracy for piling behaviour using individual-level computer-vision data from ArUco-marked laying hens
Source: Genet Sel Evol. 2026 Nov 12;58:89. doi: 10.1186/s12711-026-01109-x (PMC13760999; doi:10.1186/s12711-026-01109-x)
Supplement: Supplementary file 1 [file 12711_2026_1109_MOESM1_ESM.docx]

**Appendix A. Derivation of the cross-validation accuracy formula**

The cross-validation accuracy was estimated following the relationship between predictive ability and prediction accuracy described by [23]:

$$\text{ρ}_{\text{EBVMP}}\text{=}\frac{\text{ρ}\text{ }\left( \text{EBVMP, }\tilde{\text{y}} \right)}{\text{h}},$$

where ρ(EBVMP, $\tilde{y}$) is the correlation between the EBVMPs and the phenotypic observations corrected for fixed effects ($\tilde{y}$), and h is the square root of the trait’s heritability.

Rather than calculating ρ(EBVMP, $\tilde{y}$) directly from corrected phenotypes, we estimated it from the validation model. The model for validation phenotypes was:

$\text{y =fixed-effects + β EBVMP+ p +e }$,

where β is the regression coefficient of the validation phenotype on the EBVMP, p is the permanent environmental effect, and e is the residual effect.

The variance of validation records, conditional on the fixed effects, can therefore be expressed as:

$$\text{Var(}\tilde{\text{y}}\text{)= Var(}\text{β}\text{ EBVMP+ }\text{p }\text{+e).}$$

Because the model components are assumed to be uncorrelated, this equals:

$\text{Var}\left( \tilde{\text{y}} \right)=$ $\text{β}^{\text{2}}\text{ }\text{σ}_{\text{EBVMP}}^{\text{2}}\text{+ }\text{σ}_{\text{p}}^{\text{2}}\text{+ }\text{σ}_{\text{e}}^{\text{2}}$.

The proportion of variance in the corrected phenotype explained by the regression on EBVMP was then calculated as the coefficient of determination (R^2^).

$$\text{R}^{\text{2}}\text{= }\frac{\text{explained variance}}{\text{total variance}}$$

The explained variance is given by $\text{β}^{\text{2}}\text{* }\text{σ}_{\text{EBVMP}}^{\text{2}}$, and the total variance is given by $\text{β}^{\text{2}}\text{*} \text{σ}_{\text{EBVMP}}^{\text{2}}\text{+} \text{σ}_{p}^{\text{2}}\text{+} \text{σ}_{\text{e}}^{\text{2}}$. Thus, after substitution of these terms in R^2^ :

$\text{R}^{\text{2}}\text{= }\frac{\text{β}^{\text{2}}\text{ }\text{σ}_{\text{EBVMP}}^{\text{2}}}{\text{β}^{\text{2}}\text{ }\text{σ}_{\text{EBVMP}}^{\text{2}}\text{+ }\text{σ}_{\text{p}}^{\text{2}}\text{+ }\text{σ}_{\text{e}}^{\text{2}}}$.

For a simple linear regression, the coefficient of determination is equal to the squared correlation between the predictor and the response:

$$\text{R}^{\text{2}}\text{= }\text{ρ }^{\text{2}}\left( \text{EBVMP, }\tilde{\text{y}} \right)$$

Therefore

$$\text{ρ }\left( \text{EBVMP, }\tilde{\text{y}} \right)\text{= }\sqrt{\frac{\text{β}^{\text{2}}\text{ }\text{σ}_{\text{EBVMP}}^{\text{2}}}{\text{β}^{\text{2}}\text{ }\text{σ}_{\text{EBVMP}}^{\text{2}}\text{+ }\text{σ}_{\text{p}}^{\text{2}}\text{+ }\text{σ}_{\text{e}}^{\text{2}}}}$$

Substituting this expression into the first expression gives

$$\text{ρ}_{\text{EBVMP}}\text{=}\frac{\sqrt{\frac{{\hat{\text{β}}}^{\text{2}}\text{σ}_{\text{EBVMP}}^{\text{2}}}{{\hat{\text{β}}}^{\text{2}}\text{σ}_{\text{EBVMP}}^{\text{2}}\text{+ }\hat{\text{σ}_{\text{p}}^{\text{2}}}\text{+}\hat{\text{σ}_{\text{e}}^{\text{2}}}}}}{\text{h}}$$

which corresponds to Eq. (13) in the main text.

References:

23. Legarra A, Robert-Granié C, Manfredi E, Elsen J-M. Performance of genomic selection in mice. Genetics. 2008;180:611-8.
