# Supplementary material for: Genetic parameters and genomic prediction accuracy for piling behaviour using individual-level computer-vision data from ArUco-marked laying hens
Source: Genet Sel Evol. 2026 Nov 12;58:89. doi: 10.1186/s12711-026-01109-x (PMC13760999; doi:10.1186/s12711-026-01109-x)
Supplement: Supplementary file 2 [file 12711_2026_1109_MOESM2_ESM.docx]

**Appendix B. Derivation of between-trait prediction accuracy and genetic correlation**

The between-trait accuracy was calculated using the same approach described in Appendix A. The only difference was that the EBVMPs of one trait were used as predictors in the validation model of another trait. Therefore, the regression coefficient and the variance of the EBVMPs corresponded to the predictor trait, whereas the remaining variance components and the heritability corresponded to the target trait.

To show how the resulting between-trait accuracy is related to the genetic correlation between the two traits, let A denote the unknown true breeding value and $\hat{\text{A}}$ its predicted breeding value. We first consider the relationship between A and $\hat{\text{A}}$ . For BLUP predictions [20],

$$\text{Cov}\left( \text{A,} \hat{\text{A}} \right)\text{=} \text{σ}_{\hat{\text{A}}}^{\text{2}}$$

Now consider the regression of $\hat{\text{A}}$ on A. The regression coefficient is given by:

$\text{b}_{\hat{\text{A}}\text{,A}}\text{= }\frac{\text{Cov}\left( \hat{\text{A}}\text{, A} \right)}{\text{σ}_{\text{A}}^{\text{2}}}$ = $\frac{\text{σ}_{\hat{\text{A}}}^{\text{2}}}{\text{σ}_{\text{A}}^{\text{2}}}$.

This ratio can also be expressed in terms of prediction accuracy. Prediction accuracy, $\text{ρ}_{\hat{\text{A}}\text{,A}}$ , is the correlation between A and $\hat{\text{A}}$,

$\text{ρ}_{\hat{\text{A}}\text{,A}}\text{= }\frac{\text{Cov}\left( \hat{\text{A}}\text{, A} \right)}{\text{σ}_{\text{A}}\text{ }\text{σ}_{\hat{\text{A}}}}\text{= }\frac{\text{σ}_{\hat{\text{A}}}^{\text{2}}}{\text{σ}_{\text{A}}\text{ }\text{σ}_{\hat{\text{A}}}}\text{= }\frac{\text{σ}_{\hat{\text{A}}}}{\text{σ}_{\text{A}}}$.

Therefore,

$\text{b}_{\hat{\text{A}}\text{,A}}\text{=}$ $\text{ρ}_{\hat{\text{A}}\text{,A}}^{\text{2}}$.

Under joint normality of A and $\hat{\text{A}}$, the conditional expectation of $\hat{\text{A}}$ given A is linear and can be written as

E ($\hat{\text{A}} | \text{A}$) = $\frac{\text{Cov}\left( \hat{\text{A}}\text{, A} \right)}{\text{σ}_{\text{A}}^{\text{2}}}\text{A}$ = $\text{b}_{\hat{\text{A}}\text{,A}}\text{A}$ = $\text{ρ}_{\hat{\text{A}}\text{,A}}^{\text{2}}$A,

where breeding values are expressed as a deviation from their mean. Thus, the expected EBV for a given true breeding value is shrunk toward the population mean by a factor equal to the squared prediction accuracy.

However, individual EBVs ($\hat{\text{A}})$ can deviate from their conditional expectations. Therefore,

$\hat{\text{A}}= \text{ρ}_{\hat{\text{A}}\text{,A}}^{\text{2}}$A + e,

where e represents this deviation: e = $\hat{\text{A}}$ - E ($\hat{\text{A}} | \text{A}$). This residual should not be confused with the prediction error (A - $\hat{\text{A}}$) nor with the environmental residual. The residual e is uncorrelated with A, so Cov(A, e) = 0.

Now consider two genetically correlated traits, X and Y, with true breeding values A_X_ and A_Y_, respectively. Let X represent the predictor trait, Y the target trait, ${\hat{\text{A}}}_{\text{X}}$ the EBVs for trait X, and $\text{ρ}_{\text{X}}$ the within-trait prediction accuracy. Applying the relationship derived above to trait X:

${\hat{\text{A}}}_{\text{X}}\text{= }\text{ρ}_{\text{X}}^{\text{2}}\text{ }\text{A}_{\text{X}}\text{+}\text{e}_{\text{X}}$.

The covariance between ${\hat{\text{A}}}_{\text{X}}$ and $\text{A}_{\text{Y}}$, can be expressed as

$\text{Cov}\left( {\hat{\text{A}}}_{\text{X}}\text{, }\text{A}_{\text{Y}} \right)\text{=Cov(}\text{ρ}_{\text{X}}^{\text{2}}\text{ }\text{A}_{\text{X}}\text{+}\text{e}_{\text{X}}\text{, }\text{A}_{\text{Y}}\text{)}$.

Since the records on the target trait, Y, are not used to obtain ${\hat{\text{A}}}_{\text{X}}$, there can be no correlation between $\text{e}_{\text{X}}$ and $\text{A}_{\text{Y}}$. Thus, $\text{Cov(}\text{e}_{\text{X}}\text{, }\text{A}_{\text{Y}}\text{) =0}$. Therefore,

$\text{Cov}\left( {\hat{\text{A}}}_{\text{X}}\text{, }\text{A}_{\text{Y}} \right)\text{=}\text{ρ}_{\text{X}}^{\text{2}}\text{ }\text{Cov(}\text{A}_{\text{X}}\text{, }\text{A}_{\text{Y}}\text{)}$.

By definition,

$\text{Cov(}\text{A}_{\text{X}}\text{,} \text{A}_{\text{Y}}\text{)=} \text{r}_{\text{g}}{\text{ }\text{σ}}_{\text{A}_{\text{X}}} \text{σ}_{\text{A}_{\text{Y}}}$,

where $\text{r}_{\text{g}}$ is the genetic correlation between traits X and Y. Substituting this expression into $\text{Cov}\left( {\hat{\text{A}}}_{\text{X}}\text{, }\text{A}_{\text{Y}} \right)$ gives,

$\text{Cov}\left( {\hat{\text{A}}}_{\text{X}}\text{, }\text{A}_{\text{Y}} \right)\text{=}\text{ρ}_{\text{X }}^{\text{2}}\text{ }\text{r}_{\text{g}}\text{ }\text{σ}_{\text{A}_{\text{X}}}\text{ }\text{σ}_{\text{A}_{\text{Y}}}$.

By definition, the between-trait prediction accuracy is the correlation between ${\hat{\text{A}}}_{\text{X}}$, and $\text{A}_{\text{Y}}$:

$\text{ρ}_{{\hat{\text{A}}}_{\text{X}}\text{,}\text{A}_{\text{Y}}}\text{= }\frac{\text{Cov}\left( {\hat{\text{A}}}_{\text{X}}\text{, }\text{A}_{\text{Y}} \right)}{\text{ }\text{σ}_{{\hat{\text{A}}}_{\text{X}}} \text{σ}_{\text{A}_{\text{Y}}}}$.

Substituting the covariance derived above gives

$\text{ρ}_{{\hat{\text{A}}}_{\text{X}}\text{,}\text{A}_{\text{Y}}}\text{= }\frac{\text{ρ}_{\text{X }}^{\text{2}}\text{ }\text{r}_{\text{g}}\text{ }\text{σ}_{\text{A}_{\text{X}}}\text{ }\text{σ}_{\text{A}_{\text{Y}}}}{\text{ }\text{σ}_{{\hat{\text{A}}}_{\text{X}}} \text{σ}_{\text{A}_{\text{Y}}}}$.

As shown above, ${\text{σ}_{\text{A}_{\text{X}}}}/{\text{σ}_{{\hat{\text{A}}}_{\text{X}}}}= 1/{\text{ρ}_{\text{X}}}.$ Substitution into the expression for the between-trait prediction accuracy gives

$\text{ρ}_{{\hat{\text{A}}}_{\text{X}}\text{,}\text{A}_{\text{Y}}}\text{= }\frac{\text{ρ}_{\text{X }}^{\text{2}}\text{ }\text{r}_{\text{g}}\text{ }\text{ }\text{σ}_{\text{A}_{\text{Y}}}}{\text{ }\text{ρ}_{\text{X}} \text{σ}_{\text{A}_{\text{Y}}}}$.

After cancellation of common terms:

$\text{ρ}_{{\hat{\text{A}}}_{\text{X}}\text{,}\text{A}_{\text{Y}}}\text{= }\text{ρ}_{\text{X}}\text{r}_{\text{g}}$.

Thus, the between-trait prediction accuracy depends on both the within-trait prediction accuracy of the predictor trait and the genetic correlation between the two traits. The genetic correlation can therefore be isolated by rearranging this relationship:

$\text{r}_{\text{g}}= \frac{\text{ρ}_{{\hat{\text{A}}}_{\text{X}}\text{,}\text{A}_{\text{Y}}}}{\text{ρ}_{\text{X}}}$.

References:

20. Mrode R ,A. Linear models for the prediction of animal breeding values. 3rd ed. CABI; 2014.
